# A unified framework for spiking and gap-junction interactions in distributed neuronal network simulations

**DOI:** 10.3389/fninf.2015.00022

**Published:** 2015-09-09

**Authors:** Jan Hahne, Moritz Helias, Susanne Kunkel, Jun Igarashi, Matthias Bolten, Andreas Frommer, Markus Diesmann

**Affiliations:** ^1^Department of Mathematics and Science, Bergische Universität WuppertalWuppertal, Germany; ^2^Institute of Neuroscience and Medicine (INM-6), Institute for Advanced Simulation (IAS-6), JARA BRAIN Institute I, Jülich Research CentreJülich, Germany; ^3^Programming Environment Research Team, RIKEN Advanced Institute for Computational ScienceKobe, Japan; ^4^Simulation Laboratory Neuroscience, Bernstein Facility for Simulation and Database Technology, Institute for Advanced Simulation, Jülich Aachen Research Alliance, Jülich Research CentreJülich, Germany; ^5^Neural Computation Unit, Okinawa Institute of Science and TechnologyOkinawa, Japan; ^6^Laboratory for Neural Circuit Theory, RIKEN Brain Science InstituteWako, Japan; ^7^Department of Psychiatry, Psychotherapy and Psychosomatics, Medical Faculty, RWTH Aachen UniversityAachen, Germany; ^8^Department of Physics, Faculty 1, RWTH Aachen UniversityAachen, Germany

**Keywords:** gap junctions, waveform relaxation, supercomputer, large-scale simulation, parallel computing, spiking neuronal network

## Abstract

Contemporary simulators for networks of point and few-compartment model neurons come with a plethora of ready-to-use neuron and synapse models and support complex network topologies. Recent technological advancements have broadened the spectrum of application further to the efficient simulation of brain-scale networks on supercomputers. In distributed network simulations the amount of spike data that accrues per millisecond and process is typically low, such that a common optimization strategy is to communicate spikes at relatively long intervals, where the upper limit is given by the shortest synaptic transmission delay in the network. This approach is well-suited for simulations that employ only chemical synapses but it has so far impeded the incorporation of gap-junction models, which require instantaneous neuronal interactions. Here, we present a numerical algorithm based on a waveform-relaxation technique which allows for network simulations with gap junctions in a way that is compatible with the delayed communication strategy. Using a reference implementation in the NEST simulator, we demonstrate that the algorithm and the required data structures can be smoothly integrated with existing code such that they complement the infrastructure for spiking connections. To show that the unified framework for gap-junction and spiking interactions achieves high performance and delivers high accuracy in the presence of gap junctions, we present benchmarks for workstations, clusters, and supercomputers. Finally, we discuss limitations of the novel technology.

## 1. Introduction

Electrical synapses, or gap junctions, were classically regarded as a primitive form of neural signaling that play roles mostly in invertebrate neural circuits. Recently, advances in molecular biology revealed their widespread existence in the mammalian nervous system, such as visual cortex, auditory cortex, sensory motor cortex, thalamus, thalamic reticular nucleus, cerebellum, hippocampus, amygdala, and the striatum of the basal ganglia (Connors and Long, 2004; Hormuzdi et al., 2004), which suggests their diverse roles in learning and memory, movement control, and emotional responses (Connors and Long, 2004; Hormuzdi et al., 2004; Dere and Zlomuzica, 2011). The functional roles of gap junctions in network behavior are still not fully understood but they are widely believed to be crucial for synchronization and generation of rhythmic activity. Recent results suggest that their contribution to synchronization is versatile, as it depends on the intrinsic currents and morphology of the neurons as well as their interaction with inhibitory synapses (Hansel et al., 2012). A classification of this diversity of synchronization behaviors is addressed by the study of phase response curves (PRCs) (Mancilla et al., 2007; Coombes and Zachariou, 2009; Hansel et al., 2012), which describe a neuronal oscillator by its phase response to a perturbation. However, other prominent works also study more specific functional roles of gap junctions and combine the detailed simulation of small networks with experiments (Vervaeke et al., 2012).

Even though brain-scale neural network simulations approach the size of the brain of small primates (Herculano-Houzel, 2009) and many biological features are already included, such as the layer-specific connectivity and spike-timing dependent synaptic plasticity, simulations with correct cell densities are still lacking gap junctions. This is due to the absence of efficient algorithms to simulate gap junctions on large parallel computers. The seminal work of Pfeuty et al. (2003) investigates a network of 1600 neurons with random gap-junction coupling and an average of 10 gap junctions per neuron. The numerical integration of the entire network is done using a second-order Runge-Kutta scheme and a fixed step size of 0.01 ms. Although the approach yields accurate results it is not parallelizable and therefore not applicable to substantially larger networks. Parallelized simulators for networks of spiking neurons on the other hand suffer from a different difficulty in the handling of gap junctions: They exploit the delayed and point-event like nature of the spike interaction between neurons. In a network with only chemical synapses with delays *d*_*ij*_, the dynamics of all neurons is decoupled for the duration of the minimal delay *d*_min_ = min_*ij*_(*d*_*ij*_). The synaptic delays in networks of point-neuron models are the result of an abstraction of the axonal propagation time of the action potential and the time the postsynaptic potential needs to travel from the location of the synapse on the dendrite to the soma where postsynaptic potentials are assumed to interact. Hence, the dynamics of each neuron can be propagated independently for the duration *d*_min_ without requiring information from other neurons, such that in distributed simulations processes need to communicate spikes only after this period (Morrison et al., 2005). This delayed communication scheme is currently implemented in the NEST simulator and is crucial for its performance and scalability to supercomputers, where communication is expensive, because it is associated with a considerable latency. Gap junctions, however, are typically represented by an instantaneous interaction between pairs of neurons of the form

(1) $$I_{\text{gap},ij}(t)=g_{ij}\left(V_{i}(t)-V_{j}(t)\right).$$

This current occurs in both cells at the site of the gap junction. In point-neuron models where equipotentiality is assumed the gap-junction current immediately affects the membrane potential. This is unlike the modeling of chemical synapse in point neurons where any axonal and dendritic delays are subsumed in a retarded spike interaction. Implementing a gap junction between neuron *i* and *j* in a time-driven simulation scheme therefore requires that neuron *i* knows the membrane potential of neuron *j* and vice verse at all times. The nature of the coupling between two neurons mediated by a gap junction depends on the difference of their membrane potentials; one neuron is excited, the other one inhibited. An action potential in neuron *i* causes an excursion of the membrane potential of neuron *j* following the shape of the action potential and any subsequent after-hyperpolarization. This excursion is termed spikelet (Pfeuty et al., 2003).

**Figure 1:**
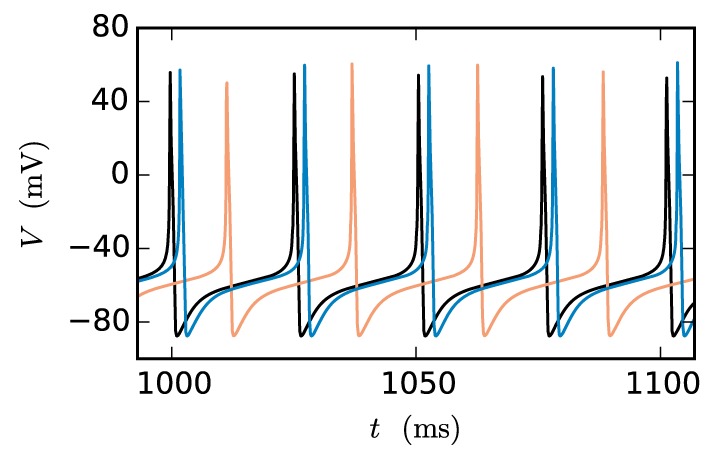
**Artefactual shift when using the single-step method**. The black curve shows the reference time course of the membrane potential of a Hodgkin-Huxley point-neuron model subject to a constant input current of 200 pA after simulating 1 s of biological time. The other curves indicate the time course of the membrane potential of the same neuron with the same input for the case that the neuron is coupled by a gap junction to a second model neuron with exactly the same properties and the simulation is carried out with the single-step approach using a Runge-Kutta-Fehlberg solver with an adaptive step-size control to cover the interval of one computation time step *h*. The orange curve displays the result for a step size of *h* = 0.1 ms and the blue curve for 0.02 ms.

At present time-driven simulators supporting gap junctions implement the instantaneous interaction with a simplification effectively decoupling the neurons for the duration of the computation time step *h*: the membrane potentials of gap-junction coupled neurons are communicated at the beginning of each time step and for the purpose of integration are assumed to be constant for the duration of the time step (for NEURON simulation software see Hines et al., 2008). There is no communication for the duration of the computation time step and no repeated communication of improved membrane potential values for a given point on the computation-time grid. In the following, we will refer to this approach as the single-step method. Note that in this framework a solver may still use adaptive step-size control to cover the interval *h*. The single-step method has two major disadvantages: Firstly communication is needed in every time step instead of in intervals of the minimal delay, which can slow down the simulation due to the latency of the employed MPI communication. Secondly the step size of the approach needs to be small. Otherwise the error results in a systematic shift of the membrane potential time course. This can be easily demonstrated by a two-neuron network: Two identical model neurons that are coupled by a gap junction should behave exactly the same as an uncoupled pair since *I*_gap_ = 0 holds at all times. However, Figure 1 shows that the single-step approach produces a significant shift within only 1 s of biological time, even when simulated with a step size that is already small compared to the time constants of the model neuron. To yield accurate results the time step would have to be exceedingly small, requiring even more communication and thereby further slowing down the simulation.

Here, we present a new iterative method based on the waveform-relaxation techniques of Lelarasmee (1982), which provides a higher accuracy than the single-step implementation while allowing us to use the delayed communication scheme. The approach can be smoothly integrated into the already existing structures of NEST as of version 2.6.0 documented in Kunkel et al. (2014). Section 2.1 describes the basics of the waveform-relaxation technique and its adaptation to the gap-junction problem. The following Section 2.2 concentrates on the technical implementation in NEST, Section 2.3 provides details on the neuron model and the used test cases, and Section 2.4 provides information on the employed computers. Section 2.5 deals with the different accuracy measures that we employed to evaluate the test cases. The results section comprises three different subsections: Sections 3.1 and 3.2 focus on the accuracy of the new iterative method in comparison to the single-step method and Section 3.3 is concerned with the performance of the gap-junction framework in NEST. Finally Section 4 discusses limitations and application areas. Preliminary results have been presented at the 7th meeting of the Brain and Neural Systems Team of the Next-Generation Integrated Simulation of Living Matter program of MEXT in September 2011 (see Diesmann, 2012 for a historical account). The technology described in the present article will be made available with one of the next major releases of NEST as open source. The conceptual and algorithmic work is a module in our long-term collaborative project to provide the technology for neural systems simulations (Gewaltig and Diesmann, 2007).

## 2. Materials and methods

### 2.1. Waveform relaxation

The waveform-relaxation methods are a set of iterative methods to solve systems of ordinary differential equations (ODEs) by dividing them into subsystems. They were derived from Picard's iterative solution of differential equations (Lumsdaine, 1992) and were originally invented by Lelarasmee (1982) for the simulation of large scale electric circuits. For any given initial value problem *y*′(*t*) = *f*(*y*(*t*)) with the initial value *y*(*t*_0_) the basic idea is to divide the ODE-system into *N* (preferably weakly coupled) subsystems

$$\begin{array}{l} y_{1}^{\prime }(t)=f_{1}(y_{1}(t),\ldots ,y_{N}(t))\\ \;\vdots \\ y_{i}^{\prime }(t)=f_{i}(y_{1}(t),\ldots ,y_{N}(t))\\ \;\vdots \\ y_{N}^{\prime }(t)=f_{N}(y_{1}(t),\ldots ,y_{N}(t)) \end{array}$$

and to solve each subsystem independently by treating the influence of the other subsystems as given input. By defining *z*_*i*_(*t*) = (*y*_1_(*t*), …, *y*_*i* − 1_(*t*), *y*_*i* + 1_(*t*), …, *y*_*N*_(*t*)) as the state of all systems except system *i* (named *d*_*i*_ in Lelarasmee et al., 1982), the subsystems can be written as

$$\begin{array}{l} y_{1}^{\prime }(t)=f_{1}(y_{1}(t),z_{1}(t))\\ \;\vdots \\ y_{i}^{\prime }(t)=f_{i}(y_{i}(t),z_{i}(t))\\ \;\vdots \\ y_{N}^{\prime }(t)=f_{N}(y_{N}(t),z_{N}(t)). \end{array}$$

Starting with an initial guess $y_{i}^{0}\left(t\right)$ for the solution of each subsystem over the entire time interval [*t*_0_, *t*_0_ + T], the solution of the original ODE-system is determined by iteratively solving the independent subsystems, where *z*_*i*_ is based on the solution of any of the previous iteration step and hence acts as a given input to the *i*-th system. The computed solutions improve step by step with each iteration.

There are several versions of waveform-relaxation methods, which differ mainly with respect to the input *z*_*i*_. Here, we employ the Jacobi waveform relaxation, where the input for the *k*-th iteration is based on the solutions of the (*k* − 1)-th iteration. For each iteration this strategy enables parallel processing of the subsystems and is hence well-suited for time-driven simulators for neuronal networks. The input *z*_*i*_ is updated at the beginning of each iteration, which results in the following subsystems for the *k*-th iteration:

(2) $$\begin{array}{l} y_{1}^{k\prime }(t)=f_{1}(y_{1}^{k}(t),z_{1}^{k-1}(t))\\ \;\vdots \\ y_{i}^{k\prime }(t)=f_{i}(y_{i}^{k}(t),z_{i}^{k-1}(t))\\ \;\vdots \\ y_{N}^{k\prime }(t)=f_{N}(y_{N}^{k}(t),z_{N}^{k-1}(t)). \end{array}$$

In order to fulfill the initial value condition, the initial guess $y_{i}^{0}\left(t\right)$ is usually chosen constant as $y_{i}^{0}\left(t\right)=y_{i}\left(t_{0}\right)\;\forall t\in \left[t_{0},t_{0}+T\right]$. For any Lipschitz-continuous function *f* the Jacobi waveform relaxation is known to converge super-linearly on every finite time interval with length T (Miekkala and Nevanlinna, 1987), such that the distance of the *k*-th iteration to the solution diminishes as

(3) $$||y^{k}-y||\leq \frac{{\left(CT\right)}^{k}}{k!}||y^{0}-y||,$$

where the constant *C* depends on the Lipschitz-constant of *f*. The Lipschitz-constant of any Lipschitz-continuous function *f* is defined as$\sup_{t_{1}\neq t_{2}}\frac{\left|f\left(t_{1}\right)-f\left(t_{2}\right)\right|}{\left|t_{1}-t_{2}\right|}$.

#### 2.1.1. Adaptation to the gap-junction problem

Conceptually, the simulation of a network of gap-junction coupled neurons amounts to the solution of a set of coupled ordinary differential equations. Unlike in simulations of networks with chemical synapses, the ODE-systems describing the behavior of two gap-junction coupled neurons *i* and *j* cannot be solved independently as their ODE-systems are connected through the *I*_gap_-term (1), which mediates a mutual and immediate influence of the corresponding membrane potentials *V*_*i*_ and *V*_*j*_ on one another. The waveform-relaxation technique allows us to solve these coupled ODE systems. While the technique is compatible with the basic optimization strategies employed in contemporary time-driven simulators such as the delayed-communication strategy described in Section 1 and the integration of the neuronal dynamics on the single-neuron level; it does require changes to the fundamental simulator structure.

Typically, the partitioning of a given ODE-system into *N* subsystems is the most critical part of the waveform-relaxation method as the coupling strength between the subsystems has a strong impact on the convergence speed. For a network of *N* gap-junction coupled neurons with membrane potentials *V*_1_, …, *V*_*N*_, however, the partitioning is already given: The ODE-system $y_{i}^{\prime }\left(t\right)=f_{i}\left(y_{i}\left(t\right),z_{i}\left(t\right)\right)$ of neuron *i* depends only on the membrane potentials of the neurons that neuron *i* connects to through gap junctions, such that the input *z*_*i*_ contains a subset of *V*_1_, …, *V*_*i*−1_, *V*_*i*+1_, …, *V*_*N*_.

#### 2.1.2. Algorithmic and numerical implementation

Applying the theoretical concept of waveform relaxation to the current problem requires (i) conveying the *k*-th approximation of the membrane potential of one neuron to another, (ii) the definition of a communication protocol, in particular the time points when information is exchanged between neurons and (iii) the definition of an error estimate of the solution used as a termination criterion. These three points are described in the current section.

##### 2.1.2.1. Approximation of the membrane potential

The Jacobi waveform relaxation method assumes that in the *k*-th iteration the values of $V_{i}^{k-1}\left(t\right)$ are known in continuous time for each subsystem. A neural simulator with a time-driven update scheme solves the system of ODEs for each neuron with an explicit numerical method with adaptive step size control, so the solution is only known on the discrete grid points which are determined by the step size *h*. We therefore need to approximate the continuous function $V_{i}^{k-1}\left(t\right)$ using the known values of the (*k* − 1)-th iteration. A discrete representation of the *k*-th approximation is moreover needed to communicate the solution to the other neurons with finite bandwidth demands. There are three obvious interpolations to consider using constant, linear or cubic interpolation between a pair of grid points. The cubic interpolation is possible, since, as we solve a system of first order differential equations, we also know the derivative $V_{i}^{\prime }=f_{i}\left(V_{i}\right)$ at each grid point. We hence have four conditions, the function value and its derivative at the left and at the right grid point, to uniquely determine the four parameters of a cubic interpolation. If we denote the interpolation of order *n*_order_ in the time interval *t* ∈ [*sh*, (*s* + 1)*h*] as

(4) $$V_{i}\left((s+x)h\right)=\sum_{m=0}^{n_{\text{order}}}a_{im}x^{m}$$

with *x* ∈ [0, 1] the coefficients of the interpolation polynomial can be determined as stated in Table 1.

**Table 1:** **Coefficients of the interpolation polynomial depending on the interpolation order**.

***n*_order_**	***a*_*i*0_**	***a*_*i*1_**	***a*_*i*2_**	***a*_*i*3_**
0	*V*_0_			
1	*V*_0_	*V*_1_ − *V*_0_		
3	*V*_0_	$hV_{0}^{\prime }$	$-3V_{0}+3V_{1}-h\left(2V_{0}^{\prime }+V_{1}^{\prime }\right)$	$2V_{0}-2V_{1}+h\left(V_{0}^{\prime }+V_{1}^{\prime }\right)$

The entries use the following abbreviation: V_0_≡V_i_(sh), V_1_≡V_i_((s+1)h), $V_{\text{0}}^{\prime }\equiv f\left(V_{i}\left(sh\right)\right)$ and $V_{\text{1}}^{\prime }\equiv f\left(V_{i}\left(\left(s+\text{1}\right)h\right)\right)$.

This scheme is independent of the details of neuronal dynamics, as long as it can be written as a system of first order differential equations. Table 2 shows the communication and storage demands generated by the use of the different approximations.

**Table 2:** **Storage and communication demand for different approximation types**.

**Approximation type**	**Number of values to…**	
	**Communicate per time step**	**Store per time step**
Constant	1	2
Linear	2	3
Cubic	4	5

The storage and communication demands only differ by the sum of the gap weights *g*_*ij*_, which needs to be stored in order to be able to sum up the incoming gap-junction connections.

The communication effort is hardly surprising, since each neuron has to communicate the coefficients of the approximating polynomial to the gap-junction coupled neurons. Even though each neuron usually has multiple gap-junction connections the incoming interpolation coefficients can be summed up. If we denote the approximation of the membrane potential *V*_*j*_ in the time interval *t* ∈ [*sh*, (*s* + 1)*h*] as in Equation (4) the total gap current $I_{gap}^{i}$ reads

(5) $$\begin{array}{l} I_{gap}^{i}=\sum_{j}g_{ij}(V_{j}-V_{i})\\ \;=-V_{i}\;\cdot \;\sum_{j}g_{ij}+\sum_{j}g_{ij}\;\cdot \;V_{j}\\ \;=-V_{i}\;\cdot \;\sum_{j}g_{ij}+\sum_{j}g_{ij}\;\cdot \;\sum_{m=0}^{n_{\text{order}}}a_{jm}x^{m}. \end{array}$$

It is obvious that within this time step the effect of all incoming gap junctions can be reduced to the *n*_order_ + 2 parameters

$${\bar{g}}_{i}=\sum_{j}g_{ij}\text{ and }{\left\{g_{i}^{m}\equiv \sum_{j}g_{ij}\;\cdot \;a_{jm}\right\}}_{m\in [0,n_{\text{order}}]}.$$

The sums appearing in *g* and *g*^0^, …, *g*^*n*_order_^ over the connected neurons *j* can hence be performed once for each iteration step and the total gap current (Equation 5) in each time step takes the form

(6) $$I_{\text{gap}}^{i}=-{\bar{g}}_{i}V_{i}+\sum_{m=0}^{n_{\text{order}}}g_{i}^{m}x^{m}.$$

**Figure 2:**
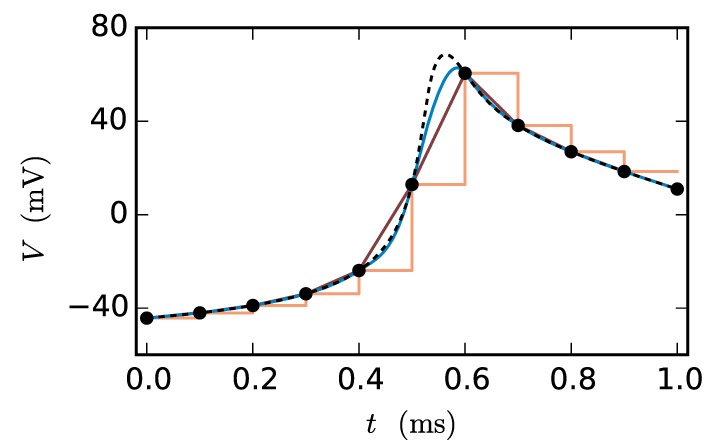
**Approximations of the membrane potential**. The dashed black curve shows the membrane potential representing an action potential (spike) and the black dots indicate the grid points used for the approximation (step size 0.1 ms). The displayed approximations are: piecewise constant (orange), linear (dark-red) and cubic (blue).

Figure 2 shows the fit of the different approximations for an exemplary course of the membrane potential. The constant interpolation is obviously a bad fit for the membrane potential, whereas the selection between linear and cubic is investigated in Section 3.1, due to the fact that the linear interpolation requires less computation and has a lower communication and storage effort. Thereby it could possibly achieve a better accuracy/simulation time trade-off.

##### 2.1.2.2. Communication strategy

**Figure 3:**
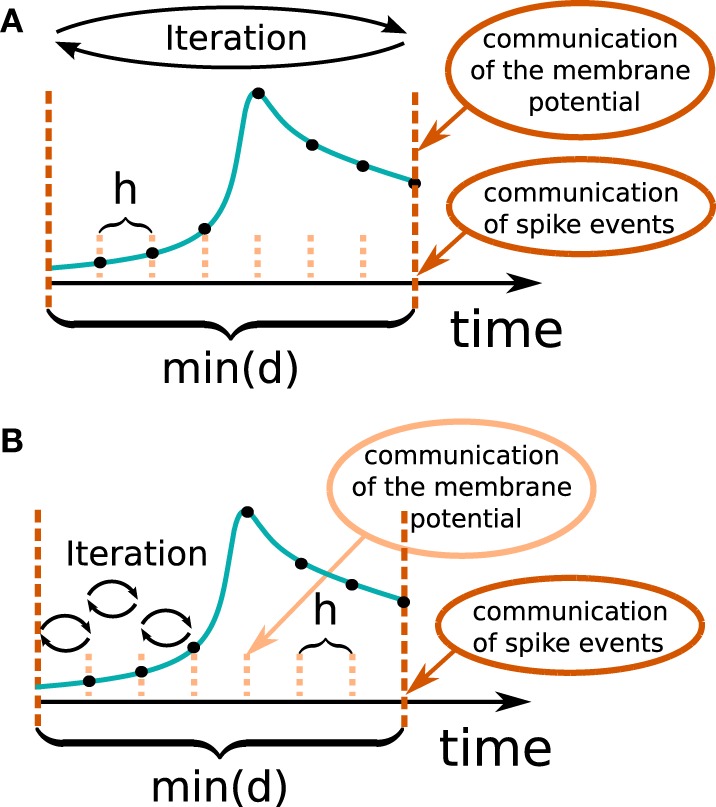
**Two communication strategies using the waveform relaxation technique in a time-driven simulator**. **(A)** The membrane potentials are communicated in intervals equal to the minimal synaptic delay. **(B)** Potentials are communicated in every computation time step *h*.

In general there are two different ways to use the waveform relaxation technique in a time-driven simulator, which are illustrated in Figure 3. The choice between the strategies is simply a question of the simulation time, since both strategies deliver nearly identical results for a given set of parameters. The uncoupling of the neurons for the duration of the minimal delay is exploited by iterating over the whole interval with length of the minimal delay, i.e. T = *d*_min_. This approach allows us to keep the benefit of only one communication per minimal delay and is therefore expected to achieve the shorter simulation time - especially for simulations on large supercomputers, where the communication latency is important. The other obvious choice for the duration of an iteration is T = *h*. Due to the faster convergence for shorter T (Equation 3), the latter choice is expected to need less iterations per time step and could for that reason be beneficial. We denote this strategy as *h*-step communication or communication in every step. Both communication strategies are investigated in Section 3.3.

##### 2.1.2.3. Iteration control

The convergence speed of the Jacobi waveform relaxation is dependent on multiple parameters. Firstly the gap weights *g*_*ij*_ have an important influence, since they determine the coupling strength between the neurons that constitute the subsystems. It is obvious that stronger interaction causes slower convergence speed, since the iterations are only needed due to the external influences. Another important influence is the duration T of the iteration interval, as suggested by Equation (3). This means that depending on the chosen communication strategy either the choice of *h* (for T = *h*) or the value of the minimal delay (for T = *d*_min_) has an influence on the number of needed iterations. As a consequence of these multiple influences the number of necessary iterations to achieve a certain accuracy may differ depending on the network to be simulated and may be hard to determine for the user of the simulator. We therefore employ an adaptive iteration control which guarantees a certain accuracy on the one hand and avoids unnecessary iterations on the other hand. We introduce a new parameter prelim_tol and stop iterating if

$$|V_{i}^{k}(t)-V_{i}^{k-1}(t)|\;\leq \text{prelim\_tol }\forall i=1,...,N$$

holds for every grid point *t*_1_, …, *t*_*m*_ within the iteration interval or the maximal number of iterations (max_num_prelim_iterations) is reached. The choice of those parameters is up to the user. The default settings are 10^−4^ for the prelim_tol and 15 for max_num_prelim_iterations. This kind of stopping criterion guarantees that if the first convergence criterion is met, further iteration would only improve the solution within the given error bound. The second criterion can be used to limit the computation time at time points that show slow convergence. If the iteration process is terminated by the second criterion the user is notified by a warning. That way the user can identify if the setting of the maximal number of iterations is adequate for the simulation. The max_num_prelim_iterations parameter should be increased if the maximal number of iterations is reached in more than a few iteration intervals. The convergence control however does not protect from inaccuracies caused by the approximation error of the interpolated membrane potential $V_{j}^{k-1}\left(t\right)$ of the respective other neurons. Therefore, the choice of *h* is also relevant for the communication strategy with T = *d*_min_, even if it does not influence the number of iterations for this particular strategy.

### 2.2. Framework for gap junctions in NEST

The waveform-relaxation method described in Section 2.1 enables the efficient numerical solution of a system of ODEs on a parallel computing system, where each of the parallel processes is responsible for a particular subsystem. It therefore constitutes a promising way of implementing continuous electrical coupling between neurons through gap junctions in distributed simulations of neuronal networks. Naturally, the method relies on inter-process communication at short time intervals to ensure that each ODE subsystem receives up-to-date information about the state of all other subsystems. As time-driven neuronal network simulators such as NEST already invoke the communication of spikes at regular intervals, it seems suitable to use these communication points to transfer also the relevant data for the waveform-relaxation method. To implement this, however, the simulator needs to provide adequate infrastructure.

The waveform-relaxation method is an iterative approach, which in the context of the presented novel gap-junction framework implies that for each communication interval, gap-junction coupled neurons need to repeatedly update their state variables until a certain accuracy criterion is fulfilled. Each iteration involves an additional communication of information about the updated state. Hence, in order to employ this method, the scheduler of the simulator needs to support the repetition of neuronal updates. Section 2.2.3 addresses this issue in more detail. Beforehand Sections 2.2.1 and 2.2.2 describe the necessary changes to the fundamental data structures and discuss the potential impact of these changes on run time and memory consumption. For the design of the novel framework we also kept in mind its potential for later extensions.

#### 2.2.1. Connection infrastructure

**Figure 4:**
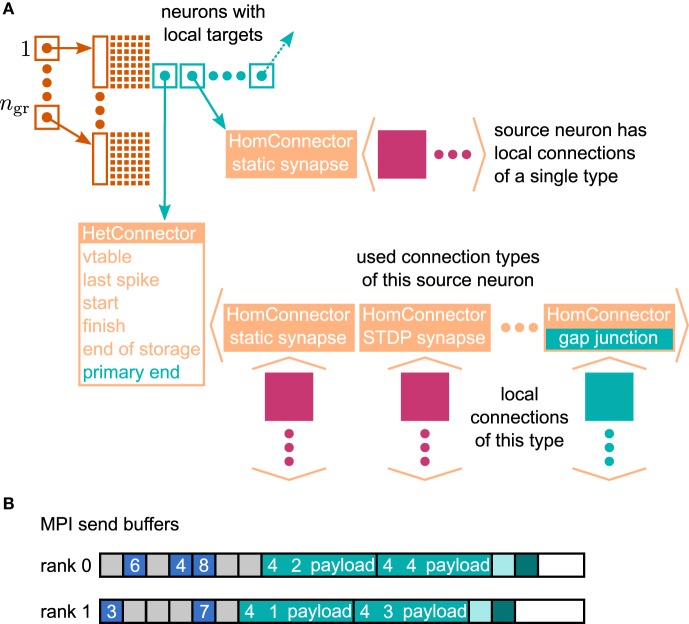
**Data structures for the representation of gap junctions**. Turquoise elements indicate necessary changes to the fundamental data structures with respect to the 4g simulation kernel of NEST (cf. Kunkel et al., 2014). **(A)** Thread-local connection infrastructure. For all neurons a sparse table (dark orange) encodes whether at least one thread-local target is present or not. If a neuron has local targets, the sparse table stores a pointer (turquoise square with arrow) to a connection container (light orange data structure), where the least significant bits of this pointer encode whether gap junctions are present or not. The container is either a HomConnector or a HetConnector depending on whether the neuron has only one or more than one type of local connection. A HomConnector directly stores the connection objects, whereas a HetConnector stores a vector of HomConnectors, one per connection type. The HomConnectors for spiking connections come first in the vector and the member primary_end is the number of spiking connection types in the vector. **(B)** MPI send buffers accumulating outgoing events in the scheduler. Toy example for a particular communication interval with two MPI processes, where rank 0 hosts the neurons with even global IDs (GIDs) and rank 1 hosts the neurons with odd GIDs. Each buffer consists of two parts: the data related to spiking connections (blue boxes) followed by the data related to gap junctions (turquoise boxes). The spike data consist of the GIDs of the local neurons that spiked in the last communication interval, where markers (light gray boxes) define the end of a simulation interval (here four simulation steps per communication step) and thereby encode the spike time. For each local neuron that has gap junctions (here neurons 1–4) the corresponding buffer contains an entry, which consists of the ID of the connection type (here gap junctions have the ID 4), the GID of the neuron, and information about the state of the neuron (payload). A marker (light turquoise box) defines the end of the gap-junction data. The final valid entry in each buffer is a boolean value (dark turquoise box), which encodes whether the local neurons require another iteration of the waveform-relaxation method. The buffers may not be completely filled (white boxes).

In the context of adaptations of the simulation kernel to current supercomputers, the connection infrastructure of NEST has undergone major changes, which reduce the memory usage. The state-of-the-art is described in Kunkel et al. (2014). In NEST, connection objects are stored on the machine that hosts the target neuron of the particular connection. The corresponding data structure is required on each thread to provide efficient access to local connection objects of a given source neuron during event delivery (filled pink and turquoise squares in Figure 4). Previously, these data structures were tailored to the delivery of spike events to local targets. The redesign presented here still supports the delivery of these primary events as described in Kunkel et al. (2012) and Kunkel et al. (2014) without compromising on performance. The delivery of data to mediate gap-junction coupling is different to the exchange of spiking activity in two respects. First, gap junctions require us to convey interpolation parameters of the membrane potentials from a sending to the receiving neuron. Second, the mechanism of data exchange should be generalizable, i.e. it should not be restricted to the implementation of gap junctions, but also applicable to other forms of interaction that require the exchange of data between neurons. The latter point implies the need to distinguish different connection types and events, called “secondary connections” and “secondary” or “payload events,” respectively. In contrast, in the following we call spiking events “primary events.” We decided for a one-to-one correspondence between a secondary synapse type and the type of secondary event that can be sent via such a connection: A secondary event of a given type will be delivered only to the targets that are connected by the matching synapse type. The concrete implementation of gap junctions requires the definition of the new connection object GapJunction that is derived from the Connection base class, as well as a class GapJEvent that is derived from SecondaryEvent.

The extended connection infrastructure shown in Figure 4A enables the storage of secondary connections. The parts of the structure that are new compared to Kunkel et al. (2014, Figure 3) are drawn in turquoise. The two main objectives of the presented design are small memory footprint and only marginal impact on the performance of the delivery of the primary spiking events. Each received primary or secondary event carries the global id (GID) of the sending neuron. The sparse table indicates at the given GID if the sending neuron has at least one (primary or secondary) connection on the local machine. That given, the sparse table provides a pointer to the attached connection containers. A spike event to be delivered is passed to all primary connections that are found below the pointer. Even though there are different connection types, such as to distinguish static connections from those exhibiting spike-timing dependent plasticity, all primary connections convey spike events. The situation is different for the secondary events, because an event containing the interpolation parameters for a gap-junction current should only be delivered to a neuron that expects this information. The latter is indicated by an existing gap-junction connection from the sending neuron. The identification between secondary events and the corresponding connection is achieved by a unique id that is assigned to each secondary synapse type and its corresponding event type upon registration at the simulation kernel.

The adaptive data structure presented in Kunkel et al. (2014) in the limit of large machines collapses along the dimension of synapse type, realized by the homogeneous connector HomConnector in Figure 4A. As a consequence, if a given source neuron only has a single target connection on a given machine or several connections of the same type, the additional infrastructure provided by the HetConnector (the linear searchable array of different connection types, the member primary_end) is not available. In this case we need a separate mechanism to decide whether or not a received primary or secondary event is to be delivered to a particular target. For reasons of performance, this decision is done in two steps. In the first check, in the case of a primary (spiking) event, we determine if the target neuron has at least one primary connection; correspondingly, for a secondary event if it has at least one secondary connection. To perform this test as early as possible and without the use of either an additional data member or the need to parse the full connection structure below the pointer, we make use of redundant information in the pointer contained in the sparse table. As pointer addresses are aligned to at least double word boundaries, their two lowest significant bits are always zero. We use the lowest significant bit to indicate whether or not the sending neuron has at least one primary connection, the second lowest significant bit to indicate the existence of at least one secondary connection. This first test can hence be done directly after retrieval of the pointer from the sparse table, which only happens if the neuron has at least one connection of any type, be it primary or secondary. To access the pointed to data structure we mask away the two lowest significant bits. The second decision depends on the container being homogeneous (containing only connections of a unique type) or heterogeneous. Delivering a primary event to a homogeneous or heterogeneous connector does not require any additional checks. The delivery of a secondary event to a homogeneous connector requires the comparison of the secondary event id to match the id of the stored connections which by definition are all the same. For heterogeneous connectors this requires a linear search in the list of secondary connections to find the connection type that matches the secondary event type, which is typically affordable as each neuron, if at all, typically only has few different incoming secondary connection types. To find the initial point of the linear search in the list of targets shown in Figure 4A, the heterogeneous connector HetConnector holds the index primary_end of the last primary connection type.

#### 2.2.2. Communication infrastructure

The payload events, introduced in Section 2.2.1 represent the path by which neurons exchange arbitrary data. In contrast to primary spiking events, that only carry the id of the sending neuron and the time stamp of event occurrence, a payload event transports additional information. We use this concept as an abstraction layer to the underlying MPI-based (Message Passing Interface Forum, 2009) data exchange. To this end, payload events support serialization of their contents into the MPI send buffers and de-serialization of these events from the MPI receive buffer. For reasons of performance, these buffers are homogeneous arrays of unsigned integers. Upon serialization, the payload event first writes out its unique type id, followed by its length as measured in multiples of unsigned integers, followed by its payload. Upon reception this process can without ambiguities be inverted, as the unique type id of the payload events allows the identification of the corresponding event type on the receiver side. Syntactically we use streaming operators (GapJEvent::operator>>(vector<unsigned int>::forward_iterator &), and the corresponding operator<<) to encapsulate the serialization and de-serialization, which requires static type casting. To avoid the duplication of data, the GapJEvent does not hold the array of coefficients for interpolation directly, but rather holds iterators to the begin and end of the corresponding coefficient arrays. On the sending side, these iterators point to the interpolation coefficients that are stored in the neuron. Upon collation of the send buffers (in function gather_events of the scheduler, see Algorithm 1), these coefficient are directly copied once from the respective neuron to the MPI send buffer. On the receiving side, the same iterators point to the positions in the receive buffer that hold the corresponding coefficients. The iterator class internally represents the positions as vector<unsigned int>>::iterator to allow fast copy into the MPI send buffers by standard algorithms (std::copy) and in addition for convenience on the side of the neuron defines an iterator interface (with functionality to increment and dereferenciation) for the represented data type, in case of the GapJEvent for double.

**Algorithm 1:** Pseudo code of the simulate function in the scheduler: Lines 9-20 show the additional code due to the new preliminary updates. An additional boolean value passed to the update function of a neuron distinguishes a preliminary update (true) from the final update (false). The gather_events() function builds the send buffer including the boolean value of the variable done, that indicates whether or not additional iterations are needed, and performs the MPI communication. The deliver_events() function distributes the received events locally and returns true only if all MPI processes indicated that the desired accuracy was achieved. The function advance_time() updates the values of *t*_*left*_ and *t*_*right*_ to the boundaries of the next time interval.

1 simulate()	2
3 […]
4
5 //*t*_left_, *t*_right_ *given*
6
7 deliver_events()
8
9 *//preliminary updates*
10 **for** *i* ∈ {1, …, max_num_prelim_iterations}:
11 *// done indicates if iteration has converged*
12 *// or more preliminary updates are needed*
13 done ← **true**
14 **for** all neuron that need prelim_update:
15 done ← update_neuron(*t*_left_, *t*_right_, **true**) && done
16
17 gather_events(done)
18 done = deliver_events()
19 **if**(done):
20 **break**
21
22 *//final update*
23 **for** all neurons :
24 update_neuron(*t*_left_, *t*_right_, **false**)
25 gather_events(**true**);
26 advance_time()
27
28 […]

Algorithm 2 shows the use of the GapJEvent in the update loop of the neuron. After the interpolation coefficients have been collected during a preliminary update, the coefficient array is passed to a newly created GapJEvent, which internally only sets the iterators accordingly, and is then sent to the network via the method send_secondary that registers the event in the scheduler. Employing the above mentioned streaming operators, upon registration secondary events are directly serialized into a separate buffer of unsigned integers for each thread. Prior to the communication step, the final send buffer is collocated by the call of the function gather_events (see Algorithm 1) by first collecting the spiking events separated by the time slices in which they occurred, as illustrated in Figure 4B. The buffers may not be completely filled as they are adapted as soon as more data needs to be transmitted but are not reduced in size in the case of fewer data. The number of time slices *d*_min_/h per communication interval of duration *d*_min_ is however fixed and the end of each time slice is marked by a special id (shown as gray square). Consequently, the receiving side knows when all spiking events have been read. Therefore, in direct succession to the spiking data the secondary events buffer for each thread is appended to the send buffer. A reserved id, invalid_id, marks the end of the secondary events, followed by a boolean value, indicating whether or not the desired accuracy has been achieved in the current iteration step, as explained in 2.2.3.

**Algorithm 2:** Update function of a neuron model supporting gap junctions. The update function acts as a time-evolution operator and propagates the state of the neuron in time from *t*_left_ to *t*_right_. The state of the neuron at time *t* is the vector **y**, where the component *y*_curr_ represents the current flowing into the membrane and **e**_curr_ is the corresponding unit vector for this component. The contribution of the gap current ${\text{e}}_{\text{curr}}\int_{t_{\text{left}}+h\left(s-1\right)}^{t_{\text{left}}+hs}I_{\text{gap}}\left(t^{\prime }\right)\;dt^{\prime }$ is given by Equation (6) and depends on the chosen method of approximation. The integral sign symbolically represents the integrator for the differential system. The component *y*_syn_ represents the component of the synaptic input current, the initial condition of which is affected by incoming synaptic impulses in line 15 and *y*_*V*_ denotes the membrane potential. The function returns if the stopping criterion is satisfied.

1	**bool** update_neuron(*t*_left_, *t*_right_, **bool** prelim_update)
2	
3	// *neuron is in state* **y**(*t*_left_)
4	
5	done ← **true**
6	*N*_coeff_ = (*n*_order_ + 1) · $\frac{t_{\text{right}}-t_{\text{left}}}{h}$
7	new_coefficients[*i*] ← 0 ∀*i* ∈{0, …, *N*_coeff_ − 1}
8	
9	**for** $s\in \{1,\ldots ,\frac{t_{\text{right}}-t_{\text{left}}}{h}\}$
10	// *propagate solution*
11	// *solve differential equation* $\frac{d}{dt}y(t)=f(y(t))+e_{\text{curr}}I_{\text{gap}}(t)$
12	// *using ḡ and $\tilde{g}$_*i*_ (see Alg. 3 for definition of $\tilde{g}$_*i*_) according to* (6)
13	**y**(*t*_left_ + *hs*) ← **y**(*t*_left_ + *h*(*s* − 1)) + $\int_{t_{\text{left}}+h(s-1)}^{t_{\text{left}}+hs}f(y(t^{\prime }))+e_{\text{curr}}I_{\text{gap}}(t^{\prime })\;dt^{\prime }$
14	
15	*y*_syn_ ← *y*_syn_ + input_buffer[*t*_lag_] // *set new synaptic input current*
16	
17	**if** (**not** prelim_update):
18	// *check for threshold and refractoriness*
19	**if** **not** refractory:
20	**if** *y_V_* > Θ:
21	emit spike
22	set neuron refractory **for** time τ_*r*_
23	**else**:
24	decrease refractory counter
25	**else**: // *preliminary update*
26	// *collect coefficients of membrane potential interpolation*
27	for *j* ∈ {0, …, *n*_order_}:
28	new_coefficients[(*s* − 1) · (*n*_order_ + 1) + *j*] ← *a*_*ij*_ // *a*_*ij*_ *as shown in (Tab. 1)*
29	// *check if stopping criterion is violated*
30	**if**( | V_last[*s* − 1] − *y*_*V*_ | ≥ prelim_tol):
31	done ← **false**
32	V_last[*s* − 1] = *y*_*V*_
33	
34	**if** (**not** prelim_update):
35	**for** $s\in \{1,\ldots ,\frac{t_{\text{right}}-t_{\text{left}}}{h}\}$:
36	new_coefficients[(*s* − 1) · (*n*_order_ + 1)] ← *y*_*V*_(*t*_right_) // *constant extrapolation*
37	V_last[*s* − 1] = 0 // *reset V_last*
38	
39	// *send interpolation coefficients to network as gap event*
40	GapJEvent ge(new_coefficients);
41	send_secondary(ge);
42	
43	// *reset data for interpolation*
44	*ḡ* ← 0
45	$\tilde{g}$_*i*_ ← 0 ∀*i* ∈ {0, …, *N*_coeff_ − 1}
46	
47	**return** done

#### 2.2.3. Iterative neuronal updates

On the scheduler level the iterative simulation of a time interval is implemented by the code lines 9-20 in Algorithm 1. Instead of just once, the update function of the involved neurons (Algorithm 2) is called several times to perform so called preliminary updates before the final update is done and the time of the simulation is advanced. The precise number of preliminary calls to the update function is determined by the iteration control as introduced in Section 2.1.2. Each neuron returns to the scheduler if its solution achieved the desired accuracy. The scheduler summarizes the feedback and sends the information to the other MPI processes. The result over all MPI processes is then returned to the scheduler to determine if a further preliminary iteration is needed.

**Figure 5:**
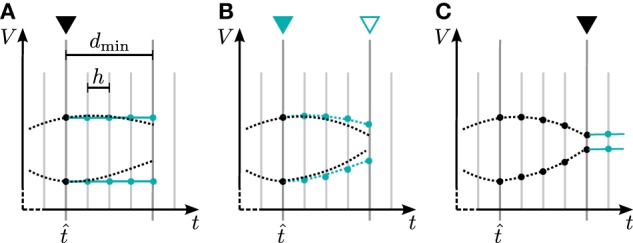
**Iterative neuronal updates**. Communication of spikes and gap-junction related data is carried out in steps of *d*_min_ (long gray lines), which denote the minimum synaptic transmission delay in the network. Within each communication interval neurons update their dynamics in steps of *h* (shorter light gray lines); here *d*_min_ = 4 h at time $\hat{t}$. Turquoise curves show the approximation of the membrane potential, which is used by the connected neuron to compute the solution in the current interval. **(A)** First iteration with constant approximation for the membrane potential of the connected neuron. At the end, a new approximation of the just computed membrane potential is passed to the connected neurons. **(B)** Further iteration with the approximation of the membrane potential from last iteration. This part is the actual iteration process which can be done multiple times. **(C)** After the final iteration a constant extrapolation for the next time step is communicated.

The discrimination between the preliminary updates and the final update is necessary, since during a preliminary update the neuron will not issue any spiking events, as shown in Algorithm 2. The incoming spiking events in each iteration are hence the same. On the other hand, only within a preliminary update a neuron will send secondary events conveying the interpolation of its membrane potential to its peers. The final, non-preliminary update conveys the extrapolation of the membrane potential to the other neurons, which will be used in the first iteration of the next time step. Figure 5 shows the realization of the iterative update process for two neurons with special focus on the communication of the interpolation coefficients. The first computation of the time step is calculated with a constant extrapolation of the membrane potential of the connected neurons. In every further iteration of the same time interval the interpolation generated with the last iteration is used. Accordingly the interpolation of the current membrane potential is computed during preliminary iterations, while for the final iteration a constant extrapolation is send to the scheduler. Thereby the interpolation coefficients are computed as described in Section 2.1.2 and saved in an array. The same applies for the receiving side (Algorithm 3), where the coefficients from the incoming connections are accumulated as described in Section 2.1.2.

**Algorithm 3:** handle function algorithm: The handle function receives the GapJEvents and collects the gap weights and interpolation coefficients according to Equation (6). In contrast to Equation (6) ${\tilde{g}}_{m+\left(s-1\right)\cdot \left(n_{\text{order}}+1\right)}:=g_{i,s}^{m}$ holds the values for all time steps *s* within one iteration interval, instead of just for one fixed time step.

1 handle(GapJEvent *e*)
2
3 //*N_coeff_ given as in (Alg. 2)*
4
5 *g* ← *g* + *e.g*
6 **for***i* ∈ {0*,…,N*_coeff_ − 1}:
7 *g_i_ ← g_i_* + *e.g* · *e.*coefficients[*i*]

The neuron update function shown in Algorithm 2 has a boolean parameter to distinguish if the current call is a preliminary or the final update. The implementation can be used with both communication strategies, since communicating in every time step (*d*_min_ = h) is only a special case and does not require further adaptation to the code.

### 2.3. Neuron and network models

#### 2.3.1. Neuron model

The neuron model used throughout the study is a point-neuron model with Hodgkin-Huxley dynamics. The model was introduced by Mancilla et al. (2007) to investigate the synchronization of electrically coupled pairs of inhibitory interneurons in neocortex. For the purpose of the present work we preferred this model over the leaky integrate-and-fire model because the former naturally includes the time course of an action potential whereas it is a point-event in the latter. The membrane potential of the model fulfills the ODE

$$\dot{V_{i}}=\frac{-I_{\text{ionic}}(V_{i},m,h,n,p)+I_{\text{ex}}+I_{\text{in}}+I_{\text{gap}}}{C_{m}}$$

with

$$\begin{array}{l} I_{\text{ionic}}=g_{\text{Na}}m^{3}h(V_{i}-V_{\text{Na}})\\ \;+\;(g_{\text{Kv3}}p^{2}+g_{\text{Kv1}}n^{4})(V_{i}-V_{\text{K}})\\ \;+\;g_{\text{leak}}(V_{i}-V_{\text{leak}})\\ \;I_{\text{gap}}=\sum_{j}g_{ij}(V_{j}-V_{i}), \end{array}$$

where the dot ˙ denotes differentiation with respect to time. The channel dynamics is given by

$$\begin{array}{l} \dot{m}=\alpha_{m}(1-m)-m\beta_{m}\\ \dot{h}=\alpha_{h}(1-h)-h\beta_{h}\\ \dot{n}=\alpha_{n}(1-n)-n\beta_{n}\\ \dot{p}=\alpha_{p}(1-p)-p\beta_{p}. \end{array}$$

A spike is transmitted to the network if the neuron passes the threshold while it is not refractory. The time of the spike is defined as the first grid point after *V*_*i*_ reaches its maximum value. Without restricting generality of the results we model synaptic events as currents described by alpha functions (see Rotter and Diesmann, 1999, Section 3.1.2). The total excitatory and inhibitory synaptic input currents

$$\begin{array}{l} I_{\text{ex}}(t)=\sum_{i=1}^{m_{\text{ex}}}J_{i}\;\cdot \;H\left(t-t_{\text{ex}}^{i}\right)\;\cdot \;e\frac{t-t_{\text{ex}}^{i}}{\tau_{\text{ex}}}\;\cdot \;\exp\left(\frac{-\left(t-t_{\text{ex}}^{i}\right)}{\tau_{\text{ex}}}\right)\\ I_{\text{in}}(t)=\sum_{i=1}^{m_{\text{in}}}J_{i}\;\cdot \;H\left(t-t_{\text{in}}^{i}\right)\;\cdot \;e\frac{t-t_{\text{in}}^{i}}{\tau_{\text{in}}}\;\cdot \;\exp\left(\frac{-\left(t-t_{\text{in}}^{i}\right)}{\tau_{\text{in}}}\right), \end{array}$$

where the *J*_*i*_ denote the synaptic weights, can be expressed by four additional first order ordinary differential equations driven by delta kicks (see Plesser and Diesmann, 2009, for a recent review) located at the points in time ${\left\{t_{ex}^{i}\right\}}_{i=1,\ldots ,m_{ex}}$ and ${\left\{t_{in}^{i}\right\}}_{i=1,\ldots ,m_{in}}$ at which the spikes arrive at the neuron. $H(x)=\{\begin{array}{ll} \begin{array}{c} 0\\ 1 \end{array} & \begin{array}{c} x<0\\ x\geq 0 \end{array} \end{array}$ denotes the Heaviside step function. The neuron model therefore consists of a system of nine ODEs. Further information on the parameters and settings can be found in Mancilla et al. (2007). The NEST implementation hh_psc_alpha_gap of the model uses the Runge-Kutta-Fehlberg solver (gsl_odeiv_step_rkf45) of the GNU Scientific Library with an adaptive step-size control (gsl_odeiv_control_y_new) to advance the state of an individual neuron by the interval *h* after which communication can occur depending on the communication strategy (Section 2.1.2) used. Thus, the solver may use finer steps to cover the interval according to the demands of the dynamics. The accuracy parameter for the absolute predicted error made in each interval h is chosen as 10^−6^, the parameter for the relative predicted error is not being used and set to 0. Therefore, there is no use in choosing the prelim_tol parameter of the waveform relaxation below the former value.

#### 2.3.2. Network models

The results in Section 3 are obtained with three different test cases. Test case 1a is used in Section 3.1 to investigate the accuracy on the single neuron level, test case 2 belongs to the network study in Section 3.2 and the Test cases 1b and 3 are investigated in Section 3.3 benchmark the performance of the framework.

**Test case 1a: pair of neurons coupled by a gap junction**. The setup consists of two hh_psc_alpha_gap neurons *i* and *j* connected by a gap junction with weight *g*_*ij*_ = 30.0 nS. Both neurons receive a constant current of 200.0 pA. All other parameters are kept at their default values (see Mancilla et al., 2007) for both neurons. The minimum delay of spike interaction is set to 1 ms.

**Test case 1b: scalable network with gap junctions only**. The Test case 1a is extended to *N* neurons to investigate the scaling of the gap-junction framework. Each neuron is coupled by gap junctions of weight *g* = 0.5 nS to 60 other neurons. For the sake of simplicity neuron *i* is coupled to the neurons from index (*i* − 30 + *N*) mod *N* to (*i* + 30) mod *N*, whereat mod denotes the modulo operator. Thus, the 30 prior and the 30 subsequent neurons if one considers the neurons aligned on a ring. All other inputs and parameters are the same as in Test case 1a.

**Test case 2: inhibitory network**. We investigate a network of 500 hh_psc_alpha_gap neurons with random initial membrane potentials between −40 and −80 mV. Each neuron receives 50 inhibitory synaptic inputs that are randomly selected from all other neurons, each with synaptic weight *J*_*I*_ = −50.0 pA and synaptic delay *d* = 1.0 ms. Each neuron receives an excitatory external Poissonian input of 500.0 Hz with synaptic weight *J*_E_ = 300.0 pA and the same delay *d*. In addition $\frac{60\cdot 500}{2}$ gap junctions are added randomly to the network resulting in an average of 60 gap-junction connections per neuron. The weight *g* of each gap-junction connection is chosen uniformly and will be varied within our tests.

**Test case 3: scalable network without gap junctions**. The setup consists of a balanced random network model (Brunel, 2000) of 80% excitatory and 20% inhibitory leaky integrate-and-fire model neurons with alpha-shaped post-synaptic currents studied by Kunkel et al. (2014) in a maximum-filling scenario. Both cell types are represented by the NEST implementation iaf_neuron with a homogeneous set of parameters. All excitatory-excitatory connections exhibit spike-timing dependent plasticity and all other synapses are static. In Kunkel et al. (2014) the network is used to characterize the differences between the 3rd and 4th generation simulation kernel of NEST. We use parameter set 1 of the former work to assess the overhead of the gap-junction framework in simulations where no gap junctions are present.

### 2.4. Computers

The results in Section 3 are obtained with three different computer systems: a workstation computer, a single shared memory node of a cluster and a distributed-memory supercomputing system. The workstation is used for the simulation of small networks investigating the accuracy of the methods (Test cases 1a and 2), while the simulations on the shared memory cluster and the supercomputer benchmark assess the scalability of the new approach in terms of run time and memory usage (Test cases 1b and 3).

The workstation computer comprises a 4-core Intel(R) Core(TM) i7-4770 processor, which runs at 3.4 GHz and supports simultaneous multithreading with 2 threads per core. 32 GB of random access memory (RAM) are available. The shared memory node of the cluster HAMBACH at the Jülich Research Centre in Germany includes compute nodes with 4 AMD Magny-Cours 12-core Opteron 6174 with 2.2 GHz and 256 GB RAM. For our study the parallelization on both systems is carried out by OpenMP (Board, 2008).

The employed supercomputer is the JUQUEEN BlueGene/Q at the Jülich Research Centre in Germany. It comprises 28, 672 nodes, each with a 16-core IBM PowerPC A2 processor, which runs at 1.6 GHz. The system supports a hybrid simulation scheme: distributed-memory parallel computing with MPI (Message Passing Interface Forum, 2009) and multithreading (OpenMP) on the processor level. 16 GB RAM are available per compute node, which are connected through a five-dimensional torus interconnect network with a bandwidth of 2 GB/s per link. In this study all benchmarks were run with 8 OpenMP threads per JUQUEEN compute node and the pool allocator (see Kunkel et al., 2014, for details). These are the same settings as in the former work, which allow us the comparison to previous results (see Section 3.3).

### 2.5. Measures of accuracy

Different measures are used to determine the accuracy of the solution. The initial two measures compare the membrane potential *V*(*t*) to a known reference solution V^*^(*t*). To demonstrate that the integration method can qualitative change the network dynamics, we also use further measures which characterize the emergent properties of the network such as firing rate and synchrony.

#### 2.5.1. Error in membrane potential time course

Firstly we employ the well-known root mean square error (RMSE)

(7) $$\epsilon =\sqrt{\frac{1}{T}\int_{0}^{T}(V^{*}(t)-V(t){)}^{2}dt},$$

which measures the deviation of the solution *V*(*t*) for the membrane potential from a reference solution V^*^(*t*) on a time interval *t* ∈ [0, T].

Since the solution is unknown in continuous time, a discrete approximation with linear interpolation between the grid points as in Henker et al. (2012) is used. This first order approximation with *N* grid points at times *t*_1_, …, *t*_*N*_ with Δ*V*_*n*_ = V^*^(*t*_*n*_) − *V*(*t*_*n*_) and Δ*t*_*n*_ = *t*_*n*+1_ − *t*_*n*_ can be determined as

(8) $$\epsilon \;\approx \;\sqrt{\frac{1}{3T}\sum_{n=1}^{N-1}\Delta t_{n}(\Delta V_{n}^{2}+\Delta V_{n+1}^{2}+\Delta V_{n}\Delta V_{n+1})}.$$

In contrast to the mean relative error measure

$$l_{2}=\frac{\sqrt{\int_{0}^{T}{\left(V^{*}(t)-V(t)\right)}^{2}dt}}{\sqrt{\int_{0}^{T}V^{*}{\left(t\right)}^{2}dt}}=\frac{\epsilon }{\sqrt{\frac{1}{T}\int_{0}^{T}V^{*}{\left(t\right)}^{2}dt}},$$

which was employed to determine the error in the membrane potential time course in Rotter and Diesmann (1999), the RMSE calculates the mean absolute error. We decided to use the latter as error measure ϵ since the behavior of the membrane potential in our test cases is well-known, which makes the absolute error a more descriptive measure.

#### 2.5.2. Temporal displacement

Secondly we introduce a measure for the shift δ between *V*(*t*) and the reference solution V^*^(*t*). The measure determines the relative time shift τ between the two signals that minimizes the RMSE

(9) $$\delta =\underset{{}_{\text{0}\leq \tau \leq \tau^{*}}}{\text{argmin}}\sqrt{\frac{\text{1}}{T}\int_{\text{0}}^{T}({\text{V}}^{*}(\text{t})-V(t+\tau ){)}^{2}dt}.$$

Of course this error measure is only reasonable, if the RMSE is indeed caused by a shift. In addition periodic signals can lead to misleading results, if, for example, there is shift of exactly one period. Nevertheless, the shift is a descriptive measure if the neurons under consideration match the required conditions. For practical usage we employ the same discretization as for the RMSE and calculate *V*(*t*_*n*_ + τ) through linear interpolation between the grid points.

#### 2.5.3. Network synchrony

For a network of *N* neurons with membrane potentials *V*_1_, …, *V*_*N*_ we determine the degree of synchrony of the network state as in Hansel et al. (2012) and Morrison et al. (2007) by the temporal fluctuations σ_*V*_ of the average membrane potential

$$V(t)=\frac{1}{N}\sum_{i=1}^{N}V_{i}(t)$$

normalized by the average temporal fluctuation σ_*V*_*i*__ of the single cells in the population. The resulting measure χ(*N*) reads

(10) $$\chi (N)=\sqrt{\frac{\sigma_{V}^{2}}{\frac{1}{N}\sum_{i=1}^{N}\sigma_{V_{i}}^{2}}}$$

and covers the interval from 0 to 1, where 1 denotes a fully synchronized network and 0 denotes the asynchronous state. The variance $\sigma_{V}^{2}$ (and analogously $\sigma_{V_{i}}^{2}$) can be calculated as

$$\sigma_{V}^{2}=\frac{1}{T}\int_{0}^{T}{\left[V(t)\right]}^{2}dt\;-\;{\left[\frac{1}{T}\int_{0}^{T}V(t)dt\right]}^{2}.$$

For our calculations the occurring integrals are approximated by the trapezoidal rule

$$\frac{1}{T}\int_{0}^{T}x(t)dt=\frac{1}{N-1}\left(\frac{x(t_{1})+x(t_{N})}{2}+\sum_{i=2}^{N-1}x(t_{i})\right).$$

#### 2.5.4. Average spike rate

For a given spike train *S*(*t*) with spikes at time *t*_1_, …, *t*_*m*_, the spike count function *n*(*t*) counting the number of spikes that have occurred up to and including time *t* can be written as

$$n(t)=\sum_{j=1}^{m}H(t-t_{j})$$

where again *H*(*x*) denotes the Heaviside step function. We determine the spike rate ν in the interval (0, T] as

$$\nu =\frac{n(T)}{T},$$

and denote the average spiking rate of a network of *N* neurons as

$$\bar{\nu }=\frac{1}{N}\sum_{i=1}^{N}\nu_{i}.$$

This estimate of the spike rate ν is consistent to the assumption that *n*(*t*) is an homogeneous Poisson process with intensity λ for which we try to estimate λ by the given realization (Kaas et al., 2014, Chapter 19).

## 3. Results

We employ three network models to study different aspects of the iterative method in comparison to the single-step method. First we investigate the pair of neurons coupled by a gap junction, which was already presented in the introduction to demonstrate the problems of the single-step method in contrast to the advanced integration schemes introduced in the present work. The approach discloses the general behavior of the methods and provides access to the single-neuron integration error not measurable in recurrent networks with chaotic dynamics (see Hanuschkin et al., 2010; Henker et al., 2012 for earlier uses of this technique). Subsequently a network of inhibitory neurons is investigated to demonstrate the simultaneous integration of spiking and gap-junction dynamics and to confirm the accuracy of the iterative method in capturing a parameter value at which a qualitative change in network activity occurs. Finally the scalable network model of Kunkel et al. (2014) is used to assess the influence of the gap-junction framework on memory consumption and simulation speed in simulations that exclusively use spiking synapses. The performance in simulations with gap junctions is measured with a scaled version of the two neuron test case. Further details on the employed test cases can be found in Section 2.3. For the remainder of the article simulation results on different hardware systems are distinguished in the figures by color: shades of green for workstations and shared memory clusters and shades of blue for the JUQUEEN BlueGene/Q at the Jülich Research Centre (see Section 2.4 for details on the employed computers).

### 3.1. Pair of gap-junction coupled neurons

We employ a pair of gap-junction coupled neurons with identical parameters and constant input current to investigate the accuracy on the single neuron level. Since both neurons behave exactly the same, their membrane voltages are identical and consequently *I*_gap_ = 0 holds at all times. Therefore, the results of a consistent gap-junction implementation should be exactly the same as for two uncoupled neurons with the same properties. In this setting the results of the uncoupled pair of neurons can be used as a reference solution to determine the quality of the investigated integration methods. The employed gap weight *g* of 30 nS represents the typical total coupling of a single neuron with the remainder of the network: the natural weight of a single gap junction is much smaller, but each neuron is connected to a couple of tens of other neurons. The test case exposes how the integration methods operate on networks of synchronized neurons coupled by gap junctions. In the absence of any chemical synapses, the minimum delay of spike interaction is set to 1 ms in order to obtain realistic results for the integration scheme that only communicates when spike times need to be exchanged. Further details on the parameters of the pair of neurons are described in Section 2.3.2 (Test case 1a).

**Figure 6:**
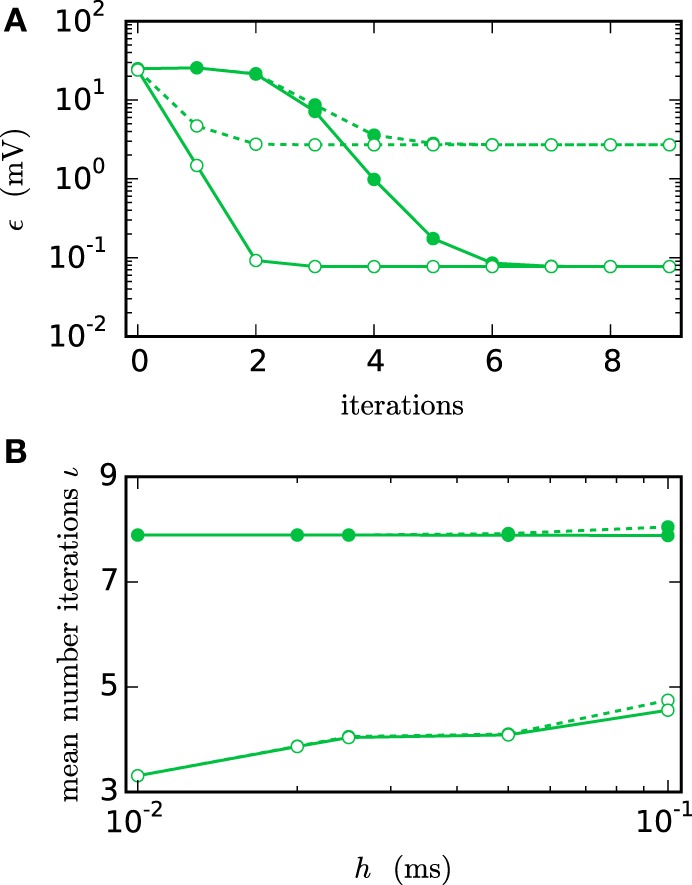
**Integration error as a function of the number of iterations**. Solid curves indicate cubic interpolation, dashed curves linear interpolation. Filled circles show results for the communication interval of NEST communication, open circles show the results for communication in every time step *h*. Color indicates the hardware system; in this and all subsequent figures shades of green represent workstations (here) or shared memory cluster node. The RMSE ϵ of the membrane potential was measured over 1 s of biological time. The step size *h* was chosen as 0.05 ms leading to $r=\frac{1.0}{0.05}=20$ time steps within one minimal delay communication interval. **(A)** RMSE for different numbers of iterations. **(B)** Mean number of iterations when using the iteration control with default settings (prelim_tol chosen as 10^−4^ and a maximum of 15 iterations, which was not reached for any simulation interval).

Figure 6A shows the functionality of the iterative method by measuring the error ϵ in the membrane potential for different numbers of iterations. The RMSE decreases with every iteration until it converges to some plateau error. The plateau error depends on the used interpolation order and is independent of the employed communication strategy. Its origin will be discussed later in Figure 8. As expected, a faster convergence is reached with the *h*-step communication, while the communication in intervals of the minimal delay takes a few more iterations. The lower panel (Figure 6B) shows the mean number of iterations when the same simulation is run with the iteration control described in Section 2.1.2. The number of needed iterations is mostly independent of the step size *h* and the used interpolation order, but differs by about four iterations for the different communication strategies.

In simulations with distributed memory the total number of communications is an important quantity, as each communication is associated with a considerable latency. If we denote with *ı*_*h*_ and *ı*_*d*_min__ the mean number of iterations with the corresponding strategy and define as $r=\frac{d_{min}}{h}$ the number of time steps per minimal delay interval, the total numbers of communications in each step (*C*_*h*_) and after each minimum delay (*C*_*d*_min__) relate to each other as

(11) $$C_{h}\approx \frac{\iota_{h}}{\iota_{d_{\text{min}}}}\;\cdot \;r\;\cdot \;C_{d_{\text{min}}}.$$

As the coupling strength of the test case relates to the total coupling of a single neuron, the simulation provides a realistic estimate for the number of iterations needed within larger network simulations. For this given estimate *C*_*h*_ exceeds *C*_*d*_min__ for *r* ≥ 3. Since *r* = 10 or *r* = 20 are more likely for an average simulation, we have *C*_*h*_ ≫ *C*_*d*_min__, so communication after each minimum delay is beneficial despite the faster convergence of the *h*-step communication strategy.

**Figure 7:**
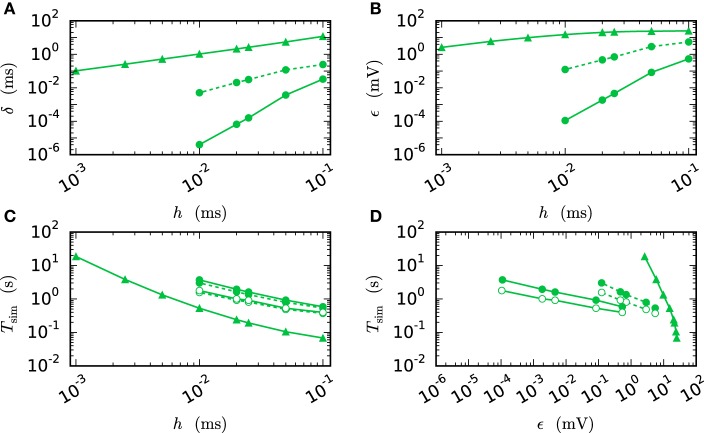
**Efficiency of a two-neuron simulation**. Triangles show results with the single-step method, while circles indicate results obtained by the iterative method. Again solid curves indicate cubic interpolation and dashed curves were obtained with linear interpolation. The used communication scheme is indicated by open (*h*-step communication) and filled symbols (communication in intervals of the minimal delay). The iteration control was used with prelim_tol chosen as 10^−6^. **(A)** Shift of the spike times after 1 s of biological time plotted against used step size *h*. **(B)** RMSE ϵ measured over 1 s of biological time plotted against the step size *h*. **(C)** Simulation time of the different approaches for 1 s of biological time. **(D)** Simulation time vs. RMSE ϵ of the corresponding simulation.

Figure 7 compares the results of the iterative method with the results of the single step methods in terms of accuracy and simulation time. Panel B measures the error ϵ of both methods for different step sizes *h*. For any given step size *h* the RMSE of the iterative method is much smaller than the RMSE of the single-step approach, which does not even reach a satisfying accuracy for step size 0.001 ms. Within the iterative method a cubic interpolation leads to a higher accuracy. Figure 7A shows that the error relates to a shift in comparison to the reference solution. This shift can be reduced up to 10^−6^ ms for the iterative method with cubic interpolation and step size 0.01 ms. At given step size *h* and leaving accuracy considerations aside, the single step method is the fastest implementation for any given step size, since no additional iterations are needed to compute the results. The iterative approach with linear interpolation saves some time in comparison to the version with cubic interpolation since less interpolation data needs to be computed and communicated. For this simple test case *h*-step communication outperforms the communication strategy in intervals of the minimal delay by a factor of 1.5, due to the very low amount of communicated data and because the communication in the employed shared memory system is fast compared to the computation. Further simulation time results for simulations on supercomputers are presented in Section 3.3. Figure 7D compares the methods in terms of efficiency. We therefore analyze the simulation time as a function of the integration error (Morrison et al., 2007), measured through the RMSE. There are two ways of reading this graph: Horizontally, one can find the most accurate method for a given simulation time. Vertically one can find the fastest method for a desired accuracy. The results show that the iterative method delivers better results in shorter time than the single step method. Also the additional effort of the cubic simulation pays off, since the method computes more accurate results in the same simulation time and reaches an accuracy which cannot be reached with the linear interpolation.

**Figure 8:**
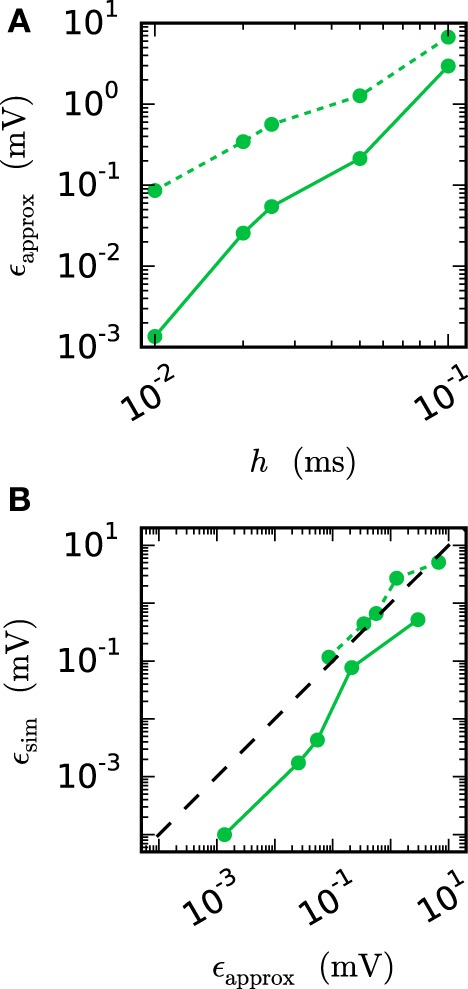
**Effect of membrane potential interpolation on network error**. **(A)** RMSE ϵ_*approx*_ of linear (dashed curves) and cubic (solid curves) interpolation for the action potential shown in Figure 2 as a function of the computation step size *h*. **(B)** Integration error for the two-neuron network (Figure 7) as a function of the interpolation error shown in **(A)**.

We observe from Figures 6 and 7 that the error of the iterative method converges to a certain plateau error that decreases with smaller step size. Figure 8 shows that the approximation of the membrane potential is the reason for this inaccuracy. The upper panel shows the error when approximating the reference spike shape in Figure 2 with different step sizes. The lower panel compares this error to the NEST RMSE when using the corresponding interpolation and same step size *h*. It turns out that the errors are basically the same, as indicated by the dotted line. The approximation error of the cubic interpolation is slightly higher than the mean simulation error, since the approximation of a spike is the most difficult part of the interpolation. The membrane potential between two spikes can be approximated almost perfectly with a cubic interpolation, although the spike shape still deviates from a cubic behavior.

### 3.2. Network with combined dynamics of chemical synapses and gap junctions

The results in Section 3.1 show the functionality of the iterative approach in purely gap-junction coupled networks. The current section investigates if the new integration method also captures the global network dynamics correctly, when both chemical synapses and gap junctions are involved, which is another important aspect for the integrator. In order to do so, we turn to population measures like the spike rate and synchrony in the network and study a network with a phase transition. Capturing the correct parameter value at which the transition occurs is a good indication that not only the single neuron error is low but also the global error. This idea and the network setting have a history in Hansel et al. (1998), Morrison et al. (2007), Coombes and Zachariou (2009), and Hansel et al. (2012). The employed network (Test case 2) consists of 500 neurons with external excitatory Poissonian input, which are coupled by inhibitory synapses and gap junctions. Without the gap junctions (meaning for *g* = 0 nS) the network shows an asynchronous irregular state (Brunel and Hakim, 1999) that is caused by the external excitatory Poissonian drive being balanced by the inhibitory feedback within the network. The network is expected to synchronize with increasing *g*. A qualitatively similar synchronization has been observed previously (Coombes and Zachariou, 2009). In this setup it is natural to use *g* as the bifurcation parameter.

**Figure 9:**
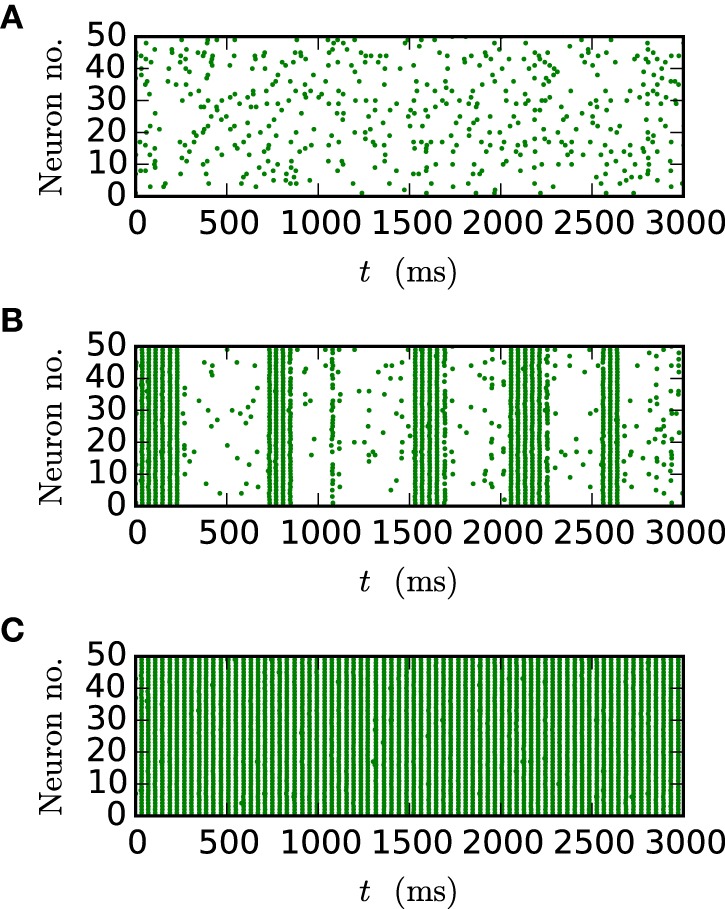
**Spike patterns for different gap weights**. The panels show the spike times of the first 50 neurons of the inhibitory network described in Section 2.3 (Test case 2) over 3 s of biological time for *J*_*I*_ = −25 pA. All results were obtained with the iterative method with cubic interpolation and step size 0.05 ms. **(A)** gap weight *g* = 0.3 nS **(B)** gap weight *g* = 0.54 nS **(C)** gap weight *g* = 0.7 nS.

**Figure 10:**
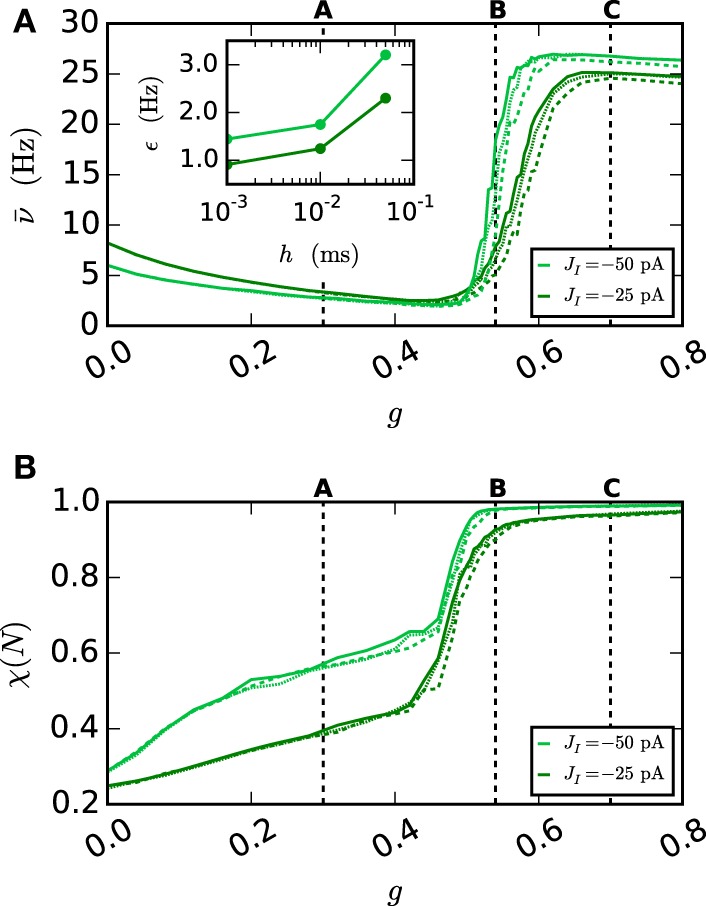
**Network behavior depending on the gap weight *g***. **(A)** The average spike rate ν and **(B)** the synchrony χ (Equation 10) of the neurons in the network, depending on the gap weight. The results for the iterative method with cubic interpolation are shown as solid curves (step size 0.05 ms) and for the single-step method with dashed (step size 0.05 ms) and dotted (step size 0.001 ms) curves. Two different synaptic amplitudes *J*_*I*_ = −50 pA and *J*_*I*_ = −25 pA were used, as indicated by the figure legend. The prelim_tol was chosen as 10^−5^ and the maximum number of iterations was not used as a stopping criterion. The simulation duration was 100 s (*J*_*I*_ = −25 pA), respectively 180 s (*J*_*I*_ = −50 pA) of biological time. The inset of (A) shows the difference between the results of the iterative method (step size 0.05 ms) and the results of the single-step method for different step sizes *h* measured by the RMSE. The dotted vertical lines correspond to the panels of Figure 9.

Figure 9 shows the spiking behavior of the employed network for different choices of the gap weight *g*. For a lower gap weight *g* = 0.3 nS the network remains in an asynchronous state. In panel B (*g* = 0.54 nS) the network switches randomly between the asynchronous to the synchronous state, while for the highest gap weight *g* = 0.7 nS a stable synchronous state is reached. The exact transition between these two states as a function of the gap weight and depending on the employed integration method is visualized in Figure 10. To overcome statistical fluctuations caused by the random transitions between the asynchronous and the synchronous state, which can be observed in Figure 9B, the system needs to be simulated for prolonged time to obtain smooth transition curves. The transition is investigated for two different choices of the synaptic weight of the inhibitory synapses to demonstrate the influence of the chemical synapses on the location of the transition. The shift of the transition point between both choices of *J*_*I*_ guarantees the influence of the chemical synapses on the global network dynamics, which is needed in order to show the correctness of the new iterative method for networks with chemical synapses and gap junctions.

As expected an increase of the gap weight leads to a higher network synchrony which also influences the spike rate. For the two choices of *J*_*I*_ the figure shows differences in the gap weight at which the network turns from the partly synchronized state to the almost fully synchronized state. In order to demonstrate the correctness of the new iterative method over the single-step method the latter is simulated for different step sizes *h*. The inset of Figure 10A shows the difference of the spike rate (measured through the RMSE) between the two methods depending on the step size. It demonstrates that the solution of the single-step method converges to the solution of the iterative method. In agreement with the results presented in Section 3.1, the convergence is slow so that even for the step size *h* = 0.001 ms the difference is still apparent.

Disregarding of the parameter value at which the transition occurs, the inaccuracy of the single-step method is also notable in the spike rate for higher gap weights (*g* > 0.6 nS), as the influence of the employed method increases with the used gap weight. The lower spike rate of the single-step method is an immediate consequence of the previously seen shift. The shift goes along with a longer distance between two spikes, which leads to the observed lower spike rate.

### 3.3. Performance of gap-junction framework in NEST

The design of the framework for gap junctions in NEST (Section 2.2) is guided by the requirements not to impair code maintainability nor to impose penalties on run time or memory usage for simulations that exclusively use chemical synapses. The first requirement is addressed by the design choice to tightly integrate the novel framework with the existing connection and communication infrastructure of NEST instead of developing an independent pathway for gap-junction related data. Thus, we are interested in the performance of (i) simulations exclusively using chemical synapses and (ii) simulations including gap junctions.

**Figure 11:**
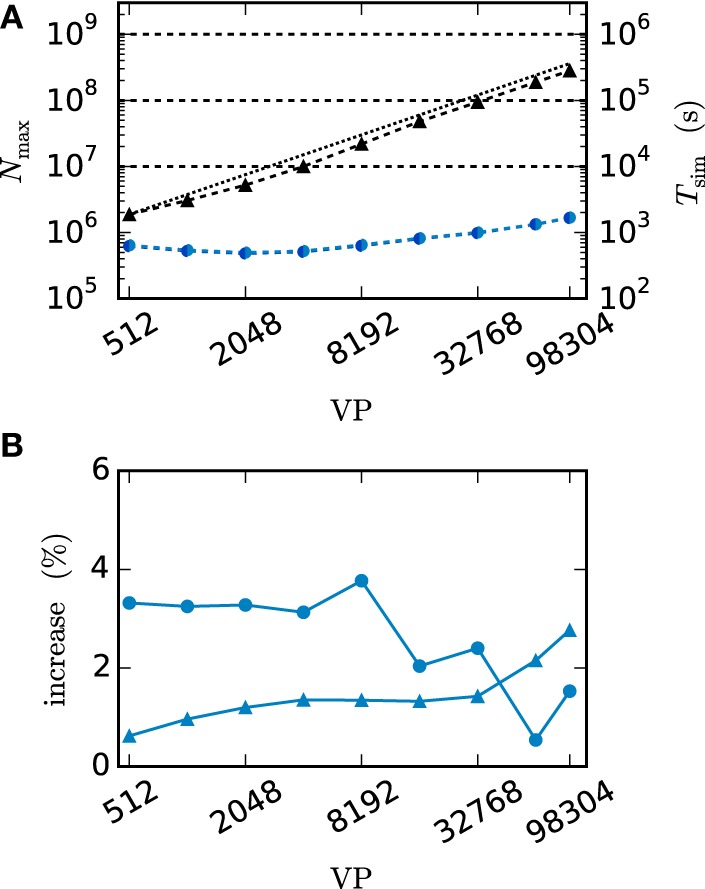
**Overhead of gap-junction framework for network with only chemical synapses**. VP denotes the overall number of processes used in line with our distribution strategy described in Section 2.4. In this and all subsequent figures shades of blue indicate the JUQUEEN supercomputer. **(A)** Triangles show the maximum network size that can be simulated in the absence of gap junctions (Test case 3). Circles show the corresponding wall-clock time required to simulate the network for 1 s of biological time. Dark blue symbols indicate the results with the 4th generation simulation kernel of NEST without the gap-junction framework and blue curves and symbols are obtained with the framework included. **(B)** Increase of time (blue circles) and memory consumption (blue triangles) due to the gap-junction framework in percent compared to the 4th generation simulation kernel.

We employ the balanced random network model (Brunel, 2000) to investigate the former issue (Test case 3) and measure the deviation in simulation time and memory usage due to the inclusion of the framework. Figure 11 shows the network in a maximum-filling scenario, where for a given machine size VP we simulate the largest possible network that completely fills the memory of the machine. Although the simulation scenario is maximum filling, we were able to simulate the same network size as before as the increase in memory usage is within the safety margin of our maximum-filling procedure (see Kunkel et al., 2014 for details on the procedure). Measured in percentage of the prior memory usage (Figure 11B) the memory consumption increases by 0.6–2.7 percent. The run time of the simulation increases by 0.5–3.8 percent. The small increase of memory usage is caused by the changes to the thread-local connection infrastructure and the communication buffer described in Sections 2.2.1 and 2.2.2. In case of primary events only (no use of gap junctions) the only extra data member is primary_end, which only affects the connection container called HetConnector. As the HetConnector is only instantiated if there are two or more synapse types targeting neurons on a given machine and having the same source neuron, this additional data member is irrelevant in the limit of large machines (sparse limit), where practically all connections are stored in HomConnectors; the latter containers only hold connections of identical types and do not have the additional data member primary_end. The small increase of the run time is due to an additional check for existence of secondary connections, which has to be done during the delivery of the events. The check is done directly after retrieving the pointer address from the sparse table and does not require additional memory as this information is encoded in redundant bits of the pointer address itself (see Section 2.2.1 for details). The reduced increase of the run time at higher numbers of virtual processes VP is due to the prolonged simulation time, as some part of the overhead is caused by the initialization in the beginning of the simulation.

Next we turn to simulations with gap junctions. The benchmarks use a scaled version of the network simulating a pair of neurons (Test case 1b), where each neuron is coupled to 60 other neurons by gap junctions. The number of neurons performing the computation and the amount of communicated data increase with *N*. We keep the conductance of a neuron accumulated over all gap junctions the same as in the case of the network comprised of a single pair (Test case 1a). As a consequence, the computations carried out by the integrator of each individual neuron are the same and hence its dynamics is independent of *N*. Thus, the performance of the gap-junction framework can be measured in a setting with fixed single neuron dynamics despite the presence of additional neurons.

**Figure 12:**
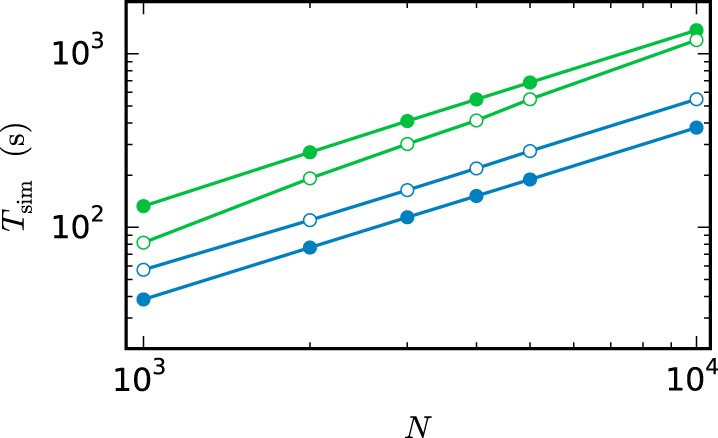
**Comparison of simulation times on different systems**. Simulation of the scaled version of the pair of neurons (Test case 1b) with different network sizes. Open symbols show the results for communication in every step (T = h) while filled symbols show the results for the original NEST communication scheme (T = *d*_min_). The simulations on the workstation (green) are executed with 8 virtual processes (8 threads). The JUQUEEN simulations (blue) use 128 virtual processes (16 MPI processes a 8 threads). 500 ms of biological time are simulated with step size *h* = 0.05 ms.

Figure 12 compares the run time of both communication strategies on JUQUEEN with their performance on workstations. On the workstation the *h*-step communication performs better due to the smaller number of iterations per interval and the fast communication through shared memory. On JUQUEEN, however, the communication in *d*_min_ steps outperforms communication in every step. As discussed in Section 3.1 the total number of communications (Equation 11) of the *h*-step communication strategy *C*_*h*_ exceeds *C*_*d*_min__. Due to the latency of the communication in a system with distributed memory the original NEST communication strategy performs better on JUQUEEN despite the comparatively small number of 16 MPI processes.

There are two major differences between simulations with and without gap junctions. Firstly, the iterative method requires the repetition of neuronal updates. Since this repetition only multiplies the run time by the number of iterations it does not affect scalability. Secondly, the approximation of the membrane potential of each neuron needs to be computed and communicated to its gap-junction coupled partners. As NEST uses MPI_Allgather to communicate data between the MPI processes, the receive buffer grows proportionally with the number of neurons. The size of the approximation data Δ of a single neuron depends on the ratio *r* between the step size *h* and the minimal delay of the network as

(12) $$\Delta =r\;\cdot \;(n_{\text{order}}+1)\;\cdot \;8\;(\text{Bytes}),$$

because each time step requires *n*_order_ + 1 double values to represent the interpolation polynomial between two adjacent time points. All interpolation coefficients are stored as 8 Bytes double variables. Consequently the number of neurons *N* is a crucial parameter for simulations with gap junctions as even in a weak-scaling scenario the local memory consumption increases with the global number of neurons. The growth is dominated by the receive buffer and effects both maximum network size and run time. This is not a particular issue of the new iterative method but rather a general property of the communication by MPI_Allgather also appearing in the single-step algorithm.

**Figure 13:**
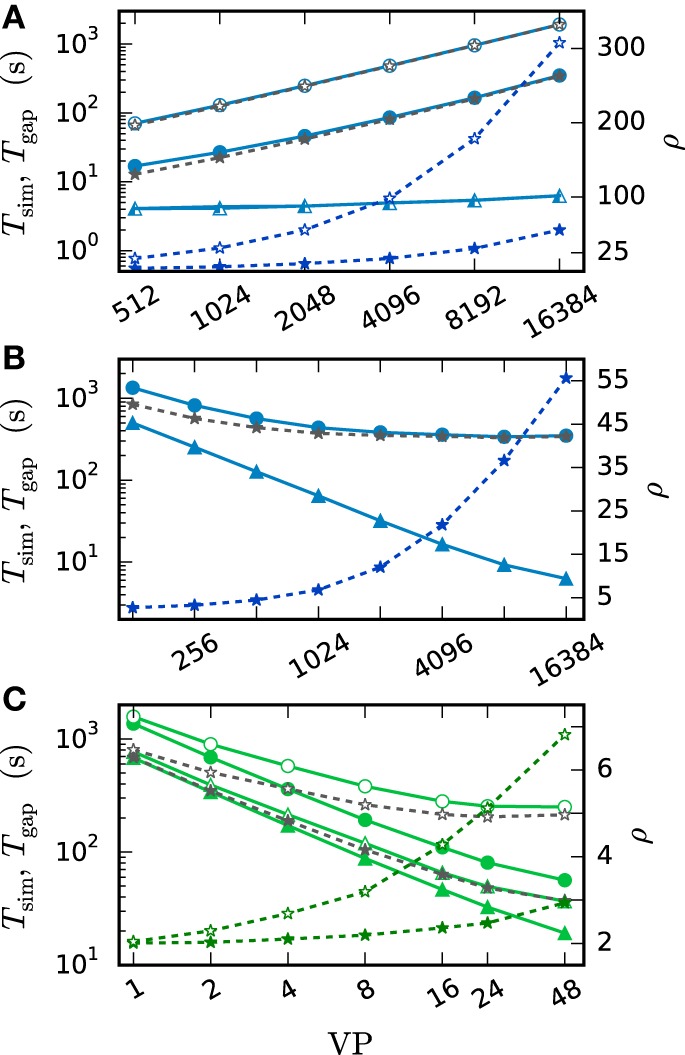
**Costs of the gap-junction dynamics**. Open symbols show the results with *h*-step communication (T = h) while filled symbols show the results with the original NEST communication scheme (T = *d*_min_, here *d*_min_ = 1 ms). The solid curves with triangles indicate the simulation time *T*_*sim*_ in the absence of gap junctions. The corresponding darker blue curves with asterisks show the ratio ρ of *T*_sim_ with and without gap junctions, while gray curves with asterisks show the difference *T*_gap_ of both simulation times. Simulations represent 50 ms of biological time for **(A,B)** and 100 ms for **(C)** at a step size of *h* = 0.05 ms. All simulations use only a single iteration per time interval. **(A)** Weak scaling of Test case 1b on JUQUEEN with *N* = 185·VP neurons. **(B)** Strong scaling of Test case 1b on JUQUEEN with *N* = 185·16384 = 3,031,040 neurons. **(C)** Strong scaling of Test case 1b run on the shared memory cluster node with *N* = 100,000 neurons.

Figure 13 investigates the slowdown due to gap-junction dynamics. This is done by simulating Test case 1b with a single iteration per time interval. The obtained results are then compared to the run time of a simulation without gap junctions but otherwise identical setup. This way the difference of the two run times *T*_gap_ can be interpreted as the time required for the additional computational load and communication. Figure 13A is a weak-scaling scenario. It demonstrates that the scalability of the method is impaired by the additional communication. Despite the constant number of neurons per virtual process and constant MPI send-buffer size the run time increases substantially. This is due to the increasing total number of neurons, which has an effect on the MPI receive buffer size and thereby on the communication time. Figure 13B studies the same setup in strong scaling with *N* ≈ 3 · 10^7^ neurons. In this scenario the receive buffer size is constant, while the size of the send buffer shrinks with increasing number of virtual processes. Here the additional time required for MPI communication is almost constant. *T*_gap_ decreases at first and then stagnates for more than 1024 virtual processes. The saturation is explained by the additional MPI-communication, which constitutes the major contribution to *T*_gap_ in this setup. As the simulation without gap junctions uses exactly the same pattern of MPI communication this is not an issue of latency but an issue of bandwidth. The initial decrease is due to the parallelization of the gap-junction dynamics: the computations on the single-neuron level, like the handling of incoming gap events, the calculation of the interpolation coefficients and their central storage in the scheduler is parallelized. In conclusion the additional time required by simulations with gap junctions on JUQUEEN is determined by the total number of neurons *N*. As the increase in run time is dominated by MPI bandwidth it cannot be eliminated by using more virtual processes VP. Therefore, it is advisable to use as few compute nodes as possible. In this setting the communication required for gap junctions increases the simulation time of one iteration for a network of *N* ≈ 3 · 10^7^ neurons by a factor of ρ = 2.5. One can multiply this factor ρ from Figure 13 with the average number of iterations *ı*_*h*_, respectively *ı*_*d*_min__ to receive an estimate of the overall increase in run time. Figure 13C shows a strong scaling scenario for a smaller network with *N* = 100,000 neurons simulated on a shared memory compute node. This setup differs from the one in panels A and B as the parallelization is implemented by OpenMP and no MPI communication is needed. Here the impact of additional virtual processes on ρ is more moderate. ρ increases from ρ = 2 for 2 threads up to ρ = 3 for 48 threads for the case where communication takes place in intervals of the minimal delay. The scalability of NEST is preserved and the time for a single iteration per time interval decreases from 1366 s with 1 thread to 56 s with 48 threads. In contrast to Figure 13B the additional time *T*_gap_ is not dominated by a constant overhead and decreases due to parallelization of the gap-junction dynamics. In the case of *h*-step communication, however, again a limit of scaling is observed. The limit is not dominated by the communication between threads but due to the serial component of event delivery in NEST; all threads inspect all incoming events.

In the following we employ the memory consumption model of Kunkel et al. (2014) to predict the maximum network size which can be simulated with both communication strategies. The model divides the overall memory into three components. M_0_(*M*) denotes the base memory usage of the simulator including external libraries such as MPI and for the sake of convenience also contains the buffers of the MPI communication. M_n_(*M*, *N*) is the additional memory usage that accrues when neurons are created and M_c_(*M*, *T*, *N*, *K*) denotes the additional memory usage that accrues when neurons are connected. The memory consumption per MPI process is thus given by

(13) $$\begin{array}{l} M(M,T,N,K)=M_{0}(M)+M_{\text{n}}(M,N)\\ \;+M_{\text{c}}(M,T,N,K), \end{array}$$

where *M* denotes the number of compute nodes, *T* the number of threads per node, *N* the number of neurons and *K* the number of synapses per neuron.

Here we extend the model to include the effect of gap junctions on memory consumption. The memory overhead of neurons with more than one local target $m_{c}^{>1}$ increases by 1 Byte due to the extra data member primary_end. The memory consumption of a connection object of type gap_junction $m_{c}^{gap}$ is the same as for a static synapse $m_{c}^{stat}$. The memory usage of a single neuron supporting gap junctions $m_{n}^{gap}$ differs from the usage of an other neuron *m*_*n*_ by 2Δ + 8*r*, as the current interpolation needs to be stored while the new interpolation coefficients are calculated (thus the factor 2) and the values from the last iteration are needed for the iteration control. In addition the base memory usage M_0_(*M*, *N*_gap_) is dependent on the number of neurons supporting gap junctions *N*_gap_ as it increases by $\left(N_{gap}+\frac{N_{gap}}{M}\right)\cdot \Delta$ due to the increases of the send and receive buffer.

**Figure 14:**
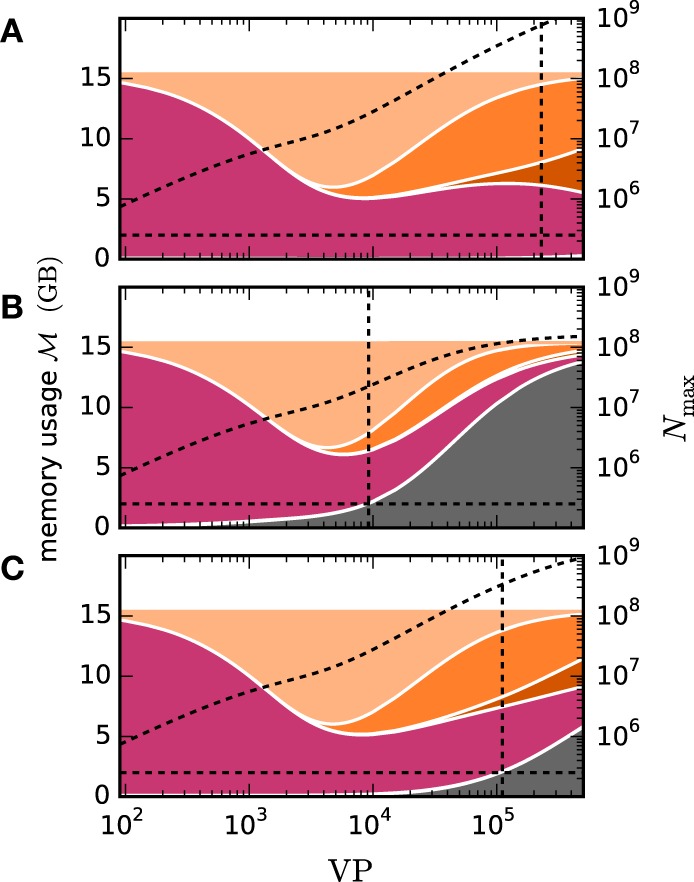
**Predicted cumulative memory usage as a function of number of virtual processes for a maximum-filling scaling**. Contributions of different data structure components to total memory usage M of NEST for Test case 3 with a network size that just fits on VP cores of JUQUEEN. The dashed black curves indicate the corresponding network size *N*_*max*_. Contributions of synapse objects and relevant components of connection infrastructure are shown in pink and shades of orange, respectively. The contribution of the base memory usage, particularly containing the receive buffer, are marked in gray. Other contributions as the neuron objects, and neuron infrastructure are significantly smaller and hence not visible at this scale. Dark orange: sparse table, orange: intermediate infrastructure containing exactly 1 synapse, light orange: intermediate infrastructure containing more than 1 synapse. The cumulative memory usage is calculated using the memory-consumption model of Kunkel et al. (2014). The horizontal dashed black line indicates 2 GB limit of a single MPI communication. Vertical lines indicate the largest number of virtual processes possible, due to full JUQUEEN, respectively exceeding a 2 GB communication buffer. **(A)** Test case 3 without gap junctions **(B)** Test case 3 with additional gap junctions between inhibitory neurons (60 gap junctions per neuron). The communication is carried out in intervals of the minimal delay (*d*_min_ = 1.5 ms and *h* = 0.1 ms) **(C)** Same setup as (B) with communication in every time step.

Figure 14 shows the contributions of gap junctions to the memory consumption under maximum filling for the network model introduced by Brunel (2000). This is Test case 3 with the addition of gap junctions between inhibitory neurons. We here use this network model to simplify the comparison to existing benchmarks (Kunkel et al., 2014). Dynamically the network model as is would not be able to support gap-junction coupling, as the leaky integrate-and-fire model (iaf_neuron) employed in this test case does not produce the shape of the action potential. Hence the interaction across gap junctions exerted by the large and positive membrane potential excursions is missing, see below for the range of neuron models available in the literature for the study of networks with gap junctions. Nevertheless, the test case provides a good estimate for the additional memory usage caused by gap junctions as the memory usages of neuron models iaf_neuron and hh_psc_alpha do not differ significantly relative to the total amount of memory consumed by chemical synapses and gap junctions. The figure shows that with increasing number of virtual processes VP the base memory component containing the MPI communication buffers becomes the dominant consumer. This is particularly apparent for communication in intervals of the minimal delay as the volume of data communicated at once is *r* times higher than for the *h*-step communication. As communication in NEST is carried out in a single MPI_Allgather call there is another relevant limit to the MPI buffer size. According to the MPI standard (Message Passing Interface Forum, 2009) the recvcount parameter counting the elements in the receive buffer is an integer value. This limits the largest possible receive buffer size to 2 GB for machines with 32 bit integer values. Therefore, the maximum network size decreases from 8·10^8^ for the case without gap junctions to 2·10^7^ for the communication in intervals of the minimal delay and to 3.5·10^8^ for the *h*-step communication. This is however not a limitation of the iterative numerical method described in this article, but a consequence of the overall communication scheme of the NEST simulation software.

## 4. Discussion

It may seem odd to discuss the integration of neuronal networks coupled by gap junctions in the context of a simulation code for which the major application area is large networks of highly simplified spiking neuron models. In these models the occurrence of an action potential is often abstracted to a threshold operation and the shape of the action potential is neglected because it has no influence on network dynamics. This changes, however, in the presence of gap junctions as the gap current depends on the difference of the membrane potentials, mediating an instantaneous coupling. The sign of the coupling depends on the time courses of the two membrane potentials. The positive excursion during an action potential of one cell exerts an excitatory drive, while the fast after-hyperpolarization immediately following the depolarization has the opposite effect. Components of the after-hyperpolarization with a longer time constant but low amplitude still have an inhibitory effect on average. The network level dynamics may not only depend on the integral effect of action potential and after-hyperpolarization but also on the time course of the interaction pattern.

Nevertheless, simulation codes designed to faithfully represent the architecture of neuronal networks typically contain a phenomenological step in their neuron models that extracts a spike time to trigger synaptic events and organize communication between the computational nodes participating in a simulation. This is independent of whether the neuron model employed is an integrate-and-fire type model or a model based on the morphological reconstruction of thousands of compartments and a detailed representation of the spiking dynamics generated by the interplay of voltage-gated ion channels. Therefore, these simulation codes have to solve the common problem of how to combine the exchange of spikes as point events with gap-junction coupling without losing the performance capability which originally motivated the design. In line with our general strategy, we use NEST for a reference implementation but the algorithm and data structures with the accompanying analysis can be transferred to other simulation codes and also to digital neuromorphic hardware like SpiNNaker (Furber et al., 2013).

The developed iterative method guarantees a high accuracy for network simulations with gap junctions regardless of the coupling strength. For networks with relatively weak coupling a sufficient accuracy can also be achieved using the less time consuming single-step method (Figure 10 before the transition phase). Here the additional expenses of the iterative method are however low, due to the integrated iteration control. For networks with sufficiently strong coupling the single-step approach causes a shift in the membrane potentials time course (Figure 1). This temporal shift reduces with the step size of the simulation. In practice, however, a researcher may firstly not be able to judge whether the coupling strength in the network model under consideration is weak enough to achieve sufficiently accurate results with the single-step method. Secondly, when the step size of the single-step approach is reduced to improve accuracy, the iterative method eventually achieves a better tradeoff between computation time and accuracy (Figure 7D).

To facilitate generalization, in the present work we use as an example a neuron model with Hodgkin-Huxley dynamics that intrinsically generates an action-potential time course and was used to study the synchronization dynamics of networks with gap junctions (Mancilla et al., 2007). However, the literature contains a range of point-neuron models suitable for interaction by gap junctions. These include alternative models of similar complexity, such as the Wang-Buzáki model (Wang and Buzsáki, 1996) that represents a fast spiking interneuron in hippocampus or cortex, but also reduced models that combine analytical tractability with the salient features of action-potential generation including a brief after-hyperpolarization, such as the absolute integrate-and-fire model (Karbowski and Kopell, 2000, reviewed in Coombes and Zachariou, 2009), and finally the exponential integrate-and-fire model (Fourcaud-Trocmé et al., 2003) and the quadratic integrate-and-fire model (Hansel and Mato, 2003) representing an intermediate level of complexity. Although the gap junction current *I*_gap_ in this study is implemented within the employed neuron model as *g*_*ij*_ (*V*_*i*_ − *V*_*j*_), the novel gap junction framework in general is able to process any form of gap junction that only depends on the involved neurons states and parameters. The necessary changes are limited to an adaptation of the neuron model and the creation of a new connection type in the hierarchy of data structures (Figure 4) to distinguish the different representations of gap junctions. A prominent example for a more complex gap junction model are voltage dependent gap junctions (Paulauskas et al., 2012). For those the optimized summation of coefficients (Equation 6) is no longer possible, resulting in a higher storage load of the single neuron. Due to the modular structure of NEST, researchers interested in understanding network dynamics can start with a detailed, possibly multi-compartmental, neuron model and then change to a more abstract and analytically tractable model while investigating the network dynamics for qualitative changes. Different parts of the network may also be described at a different level of detail.

So far simulation studies with gap junctions have only been carried out with network sizes up to a few hundred neurons and extremely simplified topology like all-to-all connectivity and only one or two cell types. These initial studies were useful to understand fundamental properties of networks with gap junctions and to verify that the new analytical tools developed are accurate. For neocortical networks this size constitutes, however, a dramatic downscaling. The number of chemical synapses per neuron is of order 10,000 and the average connection probability within a volume of a cubic millimeter where a neuron can in principle contact any other neuron is about 0.1. Thus, the minimal network size where both of these parameters can simultaneously be realized is 100,000; two orders of magnitudes larger than the networks studied up to now. The need to study neuronal networks at their natural scale has recently gained urgency by the finding that in downscaling first order measures such as spike rate can often be well preserved but already second order measures like the correlation coefficient of the spike times of two neurons are generally not preservable (van Albada et al., in press). We assume that the primary reason for the present restriction of network size found in the literature is just due to the technical difficulties in efficiently simulating larger systems and the absence of a commonly available simulation code providing such capabilities for point-neuron models. The NEURON simulation software (Carnevale and Hines, 2006) provides two multiprocessor solvers for gap junctions between electrical compartments. One incorporates the here presented single-step method using the modified Euler integration scheme into the first order backward Euler integration scheme for tree cables (Hines et al., 2008). The other uses the Sundials variable time step, variable order, ODE solver (CVODE, Cohen and Hindmarsh, 1996) to solve the global set of equations for all cells. The latter is generally too costly for large spiking network simulations as the arrival of every spike constitutes a new initial value problem. Nevertheless, the solver was successfully used in the simulation of a gap-junction coupled heart cell network (Casaleggio et al., 2014). The novel technology presented here overcomes this limitation; now networks with gap junctions can be studied at full scale. As above in the discussion of the complexity of neuron models, this does not mean that researchers have to carry out all simulations at full scale. Simulation results should just be checked with full-scale simulations to verify that they do not occur as an artifact of downscaling. The same is true for analytical results derived in the limit of infinite network size. Researchers should verify that the results hold for networks of natural size.

The additional run time costs due to the inclusion of gap junctions in an existing network simulation depend on the number of neurons of the model, as well as the kind of parallelization and the coupling strength of the gap junctions in combination with the desired accuracy. Let us look at a model of the cortical microcircuit as recently published by Potjans and Diesmann (2014) and available as open source (www.opensourcebrain.org/projects/potjansdiesmann2014). The model represents a surface area of about 1 square millimeter of cortex and has approximately the same number of neurons as the test case studied in Figure 13C. The model can easily be simulated on a single node of the compute cluster used in the present study but is time expensive because of short synaptic delays. The simulation takes 128 s with a single thread for 100 ms of biological time and about 16 s using 16 threads (data not shown). Figure 13C shows that for a network of the same size with gap junctions, using the same computational resources, and the same communication interval of 0.05 ms the time consumed by the gap-junction dynamics in a single iteration is reduced from 804 s to about 215 s. The single threaded simulations show that the additional numerical computations required for gap junctions increase run time by a factor of $\frac{128+804}{128}\cdot \iota_{h}\approx 7.3\cdot \iota_{h}$. In a realistic application the number of iterations *ı*_*h*_ required to reach the desired accuracy goal is below 5 and does not affect the scaling because no additional communication is done. At 16 threads the scaling of the network with gap junctions reaches saturation due to the large number of communication steps. Communication with a minimal delay of 1 ms reduces *T*_gap_ of a single iteration by a factor of 3.4–63 s and restores scaling. This corresponds to the reduction by a factor of 11 relative to the single threaded simulation. In conclusion networks of the size of 100,000 neurons can comfortably be simulated on a single node of a compute cluster in the presence of gap junctions. The simulation time stays within the same order of magnitude and with increasing communication interval the difference diminishes. However, looking at a single iteration in our test case using an expensive single neuron model the contribution of gap junction dynamics to the total run time increases from 51% at a single thread to 77% at 16 threads. For the simple neuron model used in the study of the cortical microcircuit the initial contribution of gap junctions is already 86% and at 16 threads reaches 93%. Thus, for the latter network model the additional costs of gap junctions are perceived as more painful.

The component limiting network size is the receive buffer of a computational node which needs to store on the order of 100 Bytes for each neuron in the network (Equation 12). With memory in the gigabyte range on a computational node, this limits network size to the order of 10 million (10^7^) neurons (see Figure 14). Thus, entire areas of the neocortex can be represented. This is promising because larger networks coupled by long-range connections should be under influence of chemical synapses only. This opens the possibility to exploit the modularity of neuronal networks in future communication algorithms to reach the brain scale.

There is still further potential of optimization. In terms of performance it might be possible to save some computation time by applying a less time consuming solver to the cell equations during the iterations because due the iterative scheme a limited accuracy may be sufficient. The benefit of such an approach is however limited, as the simulation time is mainly dominated by communication (see Figure 13). In addition the convergence theory of the waveform relaxation method assumes exact analytical solutions of the subsystems. Therefore, a high accuracy of the numerical method for the single cell equations is generally desirable as there might be an effect on the convergence speed resulting in further iterations, which would absorb the computation time savings of a less accurate solver. In terms of accuracy, given a suitable neuron model, it is possible to combine the gap junction framework with the capability of NEST to handle spike times independent of the grid spanned by the computation time step (Hanuschkin et al., 2010). A requirement on the neuron model is that an incoming synaptic impulse does neither cause a step in the membrane potential nor in its first derivative, since such discontinuities would preclude an accurate cubic approximation of the membrane potential within one time step. The neuron model studied in the manuscript satisfies these requirements, as it features alpha-shaped synaptic currents.

The framework for representing and simulating gap junctions extends the capabilities of a simulation engine for neuronal networks like NEST and widens the domain of applications. However, this comes at the price of a decrease in simulation speed by up to 3.8 percent and an increase of memory consumption of up to 2.7 percent even if no gap junctions are used. This is in contrast to the general strategy of NEST development that a researcher should only pay for features actually needed in a simulation and that a new release should not be slower or consume more memory than the previous one. The two software releases of 2014 (2.2.0 and 2.6.0) documented in Helias et al. (2012), and Kunkel et al. (2014) have concentrated on the reduction of memory consumption and also increased simulation speed. In relation to these advances the overhead of the gap-junction framework is only a minor regression; nevertheless it constitutes a nuisance future work should strive to overcome.

The limitation of the maximal network size that can be studied with the framework presented here arises from the need to communicate approximations of the membrane potential time courses between neurons. As the employed communication scheme uses collective MPI calls, these approximations are sent to all nodes that take part in the simulation irrespective of whether or not these nodes harbor neurons that require this information. This situation is qualitatively similar to the spike times being collectively communicated. However, there are two quantitative differences, the number of connections per neuron (order 10,000 vs. order 100) and the amount of information communicated (4 Byte per spike / order 100 Bytes per minimum delay). A yet more extreme scenario occurs in simulations of multi-compartment neuron models, where the approximation of the membrane potential time course of a particular compartment is relevant only for a few (order 10 down to 1) other compartments. Future work on the simulation code should assess the potential of targeted communication. Due to the low number of connections and their locality, directed communication will be particularly beneficial for gap-junction coupling.

A radically different alternative architecture was described by Kozloski and Wagner (2011) where the neuronal tissue is compartmentalized into elements of finite volume. By definition a volume element only needs to communicate with its neighbors independent of network size and parallelization is ideal.

A large body of literature already exists on the analysis of the dynamics of spiking neuronal networks. One successful route of analysis is the mapping of the spiking neuronal network to a network of nodes with continuously interacting state variables (see Bressloff, 2012, and references therein). These nodes can either represent individual neurons or populations of neurons in a mean-field sense. In both cases the result is a system of ordinary differential equations. Often instantaneously coupled systems are considered but Roxin et al. (2005) point out the importance of delays in capturing the dynamical states of the original system. In the quest for insight into the mechanisms governing network behavior, researchers routinely do further steps of simplification by linearizing effective equations that describe activity fluctuations around some steady state background activity to arrive at analytically treatable expressions. In this cascade of simplifications from the original spiking neuronal network to the analytical expression it is not always obvious under which conditions the combination of approximations hold. Therefore, researchers routinely compare simulations of spiking with rate-based models. In the absence of established tools the latter are carried out with *ad-hoc* code. Such publications do not only have to describe the implementation and numerics in detail but also the simulation experiment has to be coded twice, once for the spiking network simulator and once for the rate-based network. Thus, computational neuroscience would profit from a unique code base that features both, spiking and rate-based neuron models. The recent work by Grytskyy et al. (2013) discussing the analytical mapping of spiking network and binary networks to an effective linear rate equation including synaptic delays is an example. Future work should explore to which extend the integration framework developed here for gap junctions can also be used for networks of rate-based model neurons.

The seminal work of Hertz et al. (1991) discusses rate-based model neurons as a tool for understanding neural computation. From these origins the field of artificial neuronal networks has emerged (see Haykin, 2009, for an introduction and references) in the domain of engineering and separated from the natural sciences. The ideas are also used by researchers interested in network function and following a top-down approach. An integration framework for rate-based models embedded in a simulator for biological neuronal networks would open-up the simulation code for scientists working on functional models and facilitate the translation of ideas to spiking networks models by providing a common platform.

Recently evidence is accumulating that not only neurons are coupled by gap junctions but also astrocytes and that both networks are recurrently interacting with each other (see Giaume et al., 2010, for a review). It needs to be investigated whether the technology presented here can be generalized to the study of neuroglial interactions.

Neuroscience is still challenged by the heterogeneity of the constituents of the neuronal tissue. We hope that the progress reported here adds gap junctions as another type of brick to the Lego kit of the computational neuroscientist. The network sizes reachable with the technology described in these pages combined with the supercomputers available today now enable researchers to investigate the functional role of gap junctions in the context of an anatomically accurate circuitry.

### Conflict of interest statement

The authors declare that the research was conducted in the absence of any commercial or financial relationships that could be construed as a potential conflict of interest.
